# Effect of the Friendship Bench Intervention on Antiretroviral Therapy Outcomes and Mental Health Symptoms in Rural Zimbabwe: A Cluster Randomized Trial

**DOI:** 10.1001/jamanetworkopen.2023.23205

**Published:** 2023-07-13

**Authors:** Andreas D. Haas, Cordelia Kunzekwenyika, Josphat Manzero, Stefanie Hossmann, Andreas Limacher, Janneke H. van Dijk, Ronald Manhibi, Per von Groote, Michael A. Hobbins, Ruth Verhey, Matthias Egger

**Affiliations:** 1 Institute of Social and Preventive Medicine, University of Bern, Bern, Switzerland; 2 SolidarMed, Masvingo, Zimbabwe; 3 Clinical Trials Unit Bern, University of Bern, Bern, Switzerland; 4 SolidarMed, Luzern, Switzerland; 5 Friendship Bench, Harare, Zimbabwe; 6 Centre for Infectious Disease Epidemiology and Research, University of Cape Town, Cape Town, South Africa; 7 Population Health Sciences, Bristol Medical School, University of Bristol, Bristol, United Kingdom

## Abstract

**Importance:**

Common mental disorders (CMD), which include depression and anxiety, are prevalent among people living with HIV and are associated with suboptimal antiretroviral therapy (ART) adherence.

**Objective:**

To assess the effect of a lay health worker–led psychological intervention on ART adherence, virologic suppression, and mental health symptoms.

**Design, Setting, and Participants:**

Open-label pragmatic cluster trial with 1:1 block randomization of 16 health facilities in rural Bikita, Zimbabwe. Recruitment occurred from October 2018 to December 2019, and participants were followed up for 12 months, ending in December 2020. Participants were adults aged 18 years and older, who spoke English or Shona, screened positive for CMD (Shona Symptoms Questionnaire [SSQ]-14 score ≥9), received first-line ART for 6 or more months, had no World Health Organization stage 4 disease, no psychosis, were not pregnant, and provided informed consent. Data were analyzed from March 2021 to February 2022.

**Intervention:**

The Friendship Bench, consisting of 6 lay health worker–led weekly problem-solving therapy sessions and optional peer-led group support.

**Main Outcomes and Measures:**

The primary outcome was mean adherence during 2 to 6 months of follow-up, and the secondary outcomes were mean adherence during 1 to 12 months of follow-up, change in SSQ-14 and Patient Health Questionnaire (PHQ-9) scores (3, 6, 9, and 12 months), and viral load suppression (6 and 12 months).

**Results:**

A total of 516 participants were recruited (244 in Friendship Bench and 272 in enhanced standard care facilities); 438 (84.9%) were female and the mean (SD) age was 45.6 (10.9) years. Mean (SD) adherence between 2 to 6 months was 89.9% (18.4%) in the Friendship Bench group and 87.2% (20.1%) in the control group. The intervention had no statistically significant effect on adherence between 2 to 6 months (unadjusted mean difference, 1.93 percentage points; 95% CI, −1.20 to 5.06 percentage points; *P* = .23), between months 1 to 12 (mean difference 0.79 percentage points; 95% CI, −2.14 to 3.71 percentage points; *P* = .60), or viral suppression. Declines in SSQ-14 scores from baseline to 3 months (difference, −1.65; 95% CI, −3.07 to −0.24), 6 months (difference, −1.57; 95% CI, −2.98 to −0.15), and 9 months (difference, −1.63; 95% CI, −3.05 to −0.22) were greater in the Friendship Bench than the standard care group (*P* < .05). There were no differences in the decline in the SSQ-14 scores from baseline to 12 months and in declines in PHQ-9 scores from baseline to 3, 6, 9, and 12 months.

**Conclusions and Relevance:**

In this randomized trial of HIV-positive participants with CMD, the Friendship Bench intervention had no effect on adherence and viral suppression, possibly due to the absence of skill-based adherence training and a ceiling effect.

**Trial Registration:**

ClinicalTrials.gov Identifier: NCT03704805

## Key Points

**Question:**

Does the Friendship Bench intervention improve antiretroviral therapy (ART) adherence, viral suppression, and mental health symptoms in people living with HIV in rural Zimbabwe?

**Findings:**

In this cluster randomized trial of 516 participants, the intervention showed no significant effect on adherence to ART.

**Meaning:**

The intervention did not affect adherence, possibly due to the absence of skill-based adherence training and the ceiling effect.

## Teaser

This cluster randomized trial evaluates the effect of the Friendship Bench intervention on antiretroviral therapy outcome and mental health symptoms among patients with HIV in rural Zimbabwe.

## Introduction

Zimbabwe carries a high HIV burden, with an estimated 1.2 million people living with HIV in 2020.^1^ Antiretroviral treatment (ART) improves the life expectancy of people living with HIV,^2^ but lifelong retention and high adherence levels are vital for long-term ART effectiveness.^3,4^ The prevalence of common mental disorders (CMD), specifically depression and anxiety, among people living with HIV is high.^5,6,7^ This can be attributed to the bidirectional relationship between mental health and HIV. Mental health disorders potentially increase the risk of HIV acquisition and, conversely, living with HIV often exacerbates mental health conditions.^8^ Mental health problems can create challenges in treating HIV. They are associated with suboptimal adherence,^9,10,11^ inadequate viral suppression,^12,13,14,15,16^ low retention,^17^ and premature mortality.^16^

Treating mental disorders in people with HIV is vital for improving mental health^18,19,20,21^ and HIV management,^21,22,23,24,25^ yet mental disorders often remain undiagnosed and untreated.^26,27^ The Friendship Bench (FB), a lay health worker–led psychological intervention, was developed to increase access to treatment for CMD in Zimbabwe.^28^ The FB intervention reduced CMD symptoms in a trial in urban Zimbabwe.^28^ Its effectiveness in the rural settings and improving ART adherence and virologic suppression is unknown. This cluster randomized trial assessed the effect of the FB intervention on mental health symptoms and ART outcomes among adults living with HIV in the rural district of Bikita, where 1 in 5 adults with HIV screened positive for CMD.^7^

## Methods

### Ethics Statement

Ethics committees of the Medical Research Council of Zimbabwe, the Research Council of Zimbabwe, and the Canton of Bern, Switzerland approved the study. Individuals provided written informed consent. This report follows the Consolidated Standards of Reporting Trials (CONSORT) reporting guideline.

### Study Design

The study was a pragmatic cluster randomized open-label superiority trial in 16 health facilities in rural Zimbabwe (see Supplement 1 for study protocol and statistical analysis plan). Participants were followed up every 3 months for 1 year between October 5, 2018, and December 18, 2020.

### Setting

Bikita is a rural district about 300 km south of Harare. From 18 facilities participating in the International Epidemiology Databases to Evaluate AIDS Southern Africa (IeDEA-SA),^29^ we selected 15, excluding 3 for low patient numbers, and adding a non-IeDEA-SA site to ensure a balanced distribution of intervention and control facilities.

ART followed national treatment guidelines, except for additional CD4 testing at baseline and additional viral load testing at baseline, 6, and 12 months.^30^ Until April 2019, first-line regimens contained efavirenz or nevirapine and 2 nucleoside/nucleotide reverse transcriptase inhibitors.^30^ Subsequently, individuals receiving ART with viral loads below 1000 copies/mL were switched to a dolutegravir-based regimen. Following national treatment guidelines, participants with a viral load of more than 1000 copies/mL received enhanced adherence counseling and an additional viral load test to confirm virologic failure.^31^ See eMethods in Supplement 2 for further details.

### Randomization and Masking

We randomized 16 health facilities in a 1:1 ratio to intervention or control group. Randomization was stratified by health facility size (3 strata). All facilities were randomized at the same time using block randomization with a block size of 2 within each stratum. Treatment assignment was known to participants, clinicians, evaluators, and data analysts. Participant recruitment occurred after randomization. See eMethods in Supplement 2 for further details.

### Participants

**Figure 1.**
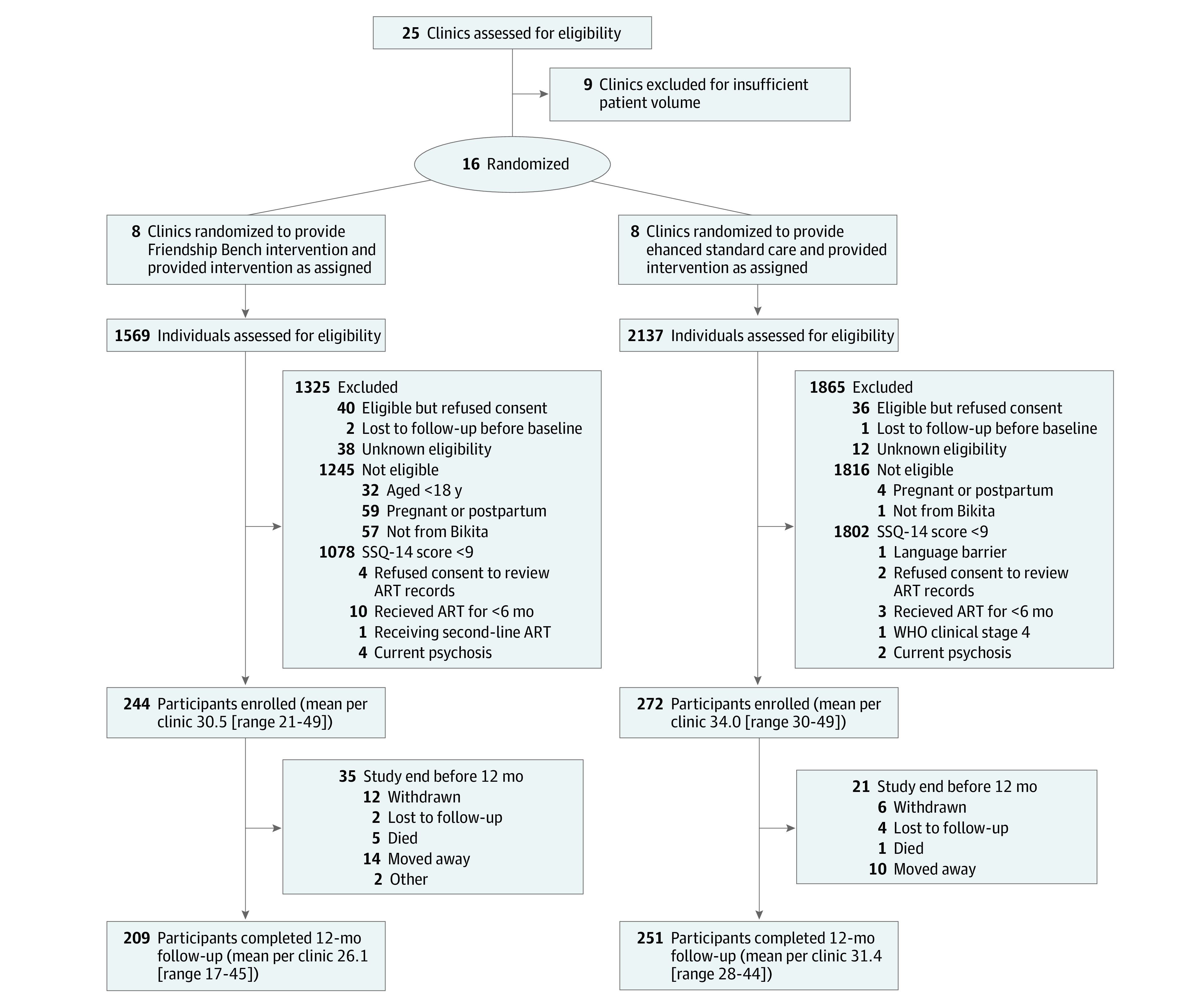
Trial Profile ART indicates antiretroviral therapy; SSQ-14, Shona Symptoms Questionnaire-14; WHO, World Health Organization.

We assessed study eligibility of individuals waiting for ART clinic appointment. Adults aged 18 years or older from Bikita who spoke English or Shona, screened positive for CMD (Shona Symptoms Questionnaire [SSQ]-14 score ≥9), had received first-line ART for at least 6 months, had no World Health Organization (WHO) stage 4 disease, no psychotic symptoms, were nonpregnant, and provided informed consent were eligible (Figure 1).

### Interventions

Participants in the intervention group were offered the FB intervention in addition to enhanced standard of care (SC). Participants in the control group received SC only.

The FB intervention consisted of 6 weekly individual counseling sessions and an optional peer-led group support. Trained lay health workers facilitated these sessions, following a structured approach to identify problems, including adherence issues, and to foster a positive attitude toward resolving them. Lay health workers were selected from the existing pool of village health workers, regardless of sex or HIV status. Two FB trainers trained lay health workers in the FB program’s manualized protocol. Clinic nurses, trained in FB supervision, regularly supervised lay health workers. The intervention was delivered on a park bench at the health facilities or in clinic consultation rooms, making it easily accessible for individuals.

After 4 sessions, participants were invited to join a peer-led group activity where they were trained in income-generating skills (eg, producing bags from recycled plastic). The support groups were initiated and facilitated by the lay health workers who provided the individual counseling sessions. These groups offered continuous support from lay health workers and peers. The FB intervention is described in detail elsewhere.^28,32^

SC consisted of nurse-led brief counseling, education regarding CMD, prescription of an antidepressant (fluoxetine), or referral to a psychiatric facility if indicated (see eMethods in Supplement 2). The nurses were trained in managing mental, neurological, and substance use disorders.^33^

### Measures

We used the Medication Event Monitoring System (MEMS) electronic pill box (AARDEX) to measure adherence. We calculated monthly mean adherence as the percentage of days participants opened the box once or twice (depending on the regimen). We treated adherence of less than 10% as missing, assuming participants did not use their MEMS device. We assessed self-reported baseline adherence according to 30-day recall. eMethods in Supplement 2 provides further details.

Research assistants administered the SSQ-14^34,35^ and the Patient Health Questionnaire (PHQ-9)^35,36^ at baseline and the 3, 6, 9, and 12-month visits. The SSQ-14 is a locally developed brief screening tool to assess symptoms of depression and anxiety in the Shona language in Zimbabwe. The tool evaluates symptoms including overthinking, difficulties in concentration, irritability, stomachache, sleep disturbance, suicidal ideations, tearfulness, perceptual symptoms, and impaired functioning in the past week,^35^ with the score ranging from 0 (no symptoms) to 14 (all symptoms present).^34^ The tool was developed through ethnographic and qualitative research, which elicited idioms of distress related to mental disorders. With a cutoff score of 9 or more, the tool has demonstrated 88% sensitivity and 76% specificity for detecting depression or anxiety among people with HIV in urban Zimbabwe.^35^ The PHQ-9 is a screening tool for depression symptoms over the past 2 weeks. A score of 11 or more has 88% sensitivity and 71% specificity for detecting depression in people with HIV in urban Zimbabwe.^35^ At baseline, research assistants administered structured questionnaires on participants’ sociodemographic characteristics, perceived general health perception, ART knowledge, alcohol use (Alcohol Use Disorders Identification Test C [AUDIT-C]^37^), food insecurity (Household Food Insecurity Scale), and social support (Medical Outcomes Study Social Support Survey [MOS-SS]).

### Outcomes

The primary outcome was mean adherence between 2 to 6 months of follow-up. Secondary outcomes included mean adherence between 1 to 12 months; change from baseline SSQ-14 and PHQ-9 score at 3, 6, 9, and 12 months; and change in viral suppression (viral load <1000 copies per mL) at months 6 and 12; and positive screening for CMD (SSQ-14 score ≥9) and depression (PHQ-9 score ≥11) at 3, 6, 9, and 12 months.

### Sample Size

The study was powered to detect an absolute 10% difference in mean adherence^24^ between months 2 to 6, assuming a standard deviation of 20, an intracluster coefficient of 0.05, and a type I error of 5% (see Supplement 1). Sixteen clusters with 25 participants provide 92% power to detect such a difference. To allow for attrition, we set the target sample size at 480 participants (16 clusters with 30 participants).

### Statistical Analysis

The primary analysis was per intention to treat. We first calculated participants’ monthly mean adherence scores and then analyzed these scores using linear mixed-effects models to assess the difference in mean adherence. Models included a random intercept and slope to account for the correlation within participants. A random intercept accounted for the clustering of individuals in health facilities, indicators defined treatment assignment, the month of analysis time (categorical), and interactions between the 2. Subsequently, we calculated the average difference in mean adherence between months 2 to 6 and 1 to 12 based on contrasts. We estimated odds ratios (ORs) for the difference in the proportion of participants with viral suppression at 6 and 12 months using logistic mixed-effect models. We conducted prespecified adjusted analyses of mean adherence and viral suppression, controlling for facility size, age, and sex. In post hoc sensitivity analyses, we adjusted for facility size, age, sex, self-reported baseline adherence, baseline SSQ-14 score, ART regimen, PHQ-9 score, WHO clinical stage, CD4 cell count, viral suppression, AUDIT-C score, MOS-SS score, and travel cost. We also assessed the difference in change from baseline in SSQ-14 and PHQ-9 scores using the same linear mixed-effects model as described previously but adjusted for the baseline score. We used the logistic mixed-effect models described previously to assess the difference in the proportion of participants with an SSQ-14 score of 9 or higher and a PHQ-9 score of 11 or higher at 3, 6, 9, and 12 months. We repeated analyses of primary and secondary outcomes using a per-protocol analysis, excluding participants who did not receive the allocated intervention and those with missing adherence data between months 2 to 6. Missing data were imputed using multiple imputation by chained equations.^38^ We used linear and logistic mixed effects models. All tests were 2-sided, and we considered a *P* value of less than .05 as statistically significant. Statistical analyses were completed in Stata, version 16 (StataCorp). eMethods in Supplement 2 provides further details. Data were analyzed from March 2021 to February 2022.

## Results

### Participants and Baseline Characteristics

In this study, 516 individuals (mean [SD] age 45.6 [10.9] years; 438 [84.9%] female) were included, with 244 participants in 8 clusters in the FB group and 272 participants in 8 clusters in the control group (Table 1). Most participants were married or cohabitating (265 participants [51.4%]) and had completed primary (229 participants [44.4%]) or secondary education (256 participants [49.6%]). The mean (SD) SSQ-14 score at baseline was 10.0 (1.1), and the mean (SD) PHQ-9 was 7.7 (3.5). The median (IQR) baseline CD4 cell count was 552 (401-721) cells/μL, few participants (29 participants [5.6%]) had a viral load of 1000 copies/mL or more, and most (405 participants [78.5%]) reported optimal adherence (ie, not having missed a single dose) in the 30 days before enrollment. Participants in the intervention group had a higher baseline mean PHQ-9 score, were more likely to be in WHO clinical stage 1, had lower MOS-SS and AUDIT-C scores, and had lower transportation costs than participants in the control group (Table 1).

**Table 1.** Sociodemographic and Clinical Characteristics of Participants at Baseline

Characteristic	Patients, No. (%)		
	Friendship Bench (n = 244)	Standard of care (n = 272)	Total (N = 516)
Age, mean (SD), y	45.6 (11.3)	45.5 (10.5)	45.6 (10.9)
Sex			
Female	211 (86.5)	227 (83.5)	438 (84.9)
Male	33 (13.5)	45 (16.5)	78 (15.1)
Marital status			
Married/living together	119 (48.8)	146 (53.7)	265 (51.4)
Widowed/divorced/separated	111 (45.5)	120 (44.1)	231 (44.8)
Single	13 (5.3)	6 (2.2)	19 (3.7)
Missing	1 (0.4)	0	1 (0.2)
Education			
Primary	114 (46.7)	115 (42.3)	229 (44.4)
Secondary or higher	110 (45.1)	146 (53.7)	256 (49.6)
Missing	20 (8.2)	11 (4.0)	31 (6.0)
SSQ-14 score, mean (SD)^a^	10.1 (1.1)	9.9 (1.1)	10.0 (1.1)
PHQ-9 score, mean (SD)^b^	8.3 (3.6)	7.2 (3.2)	7.7 (3.5)
Missing	1 (0.4)	0	1 (0.2)
Time on ART, median (IQR), y	7.9 (5.0-9.7)	6.6 (4.4-8.6)	7.0 (4.8-9.1)
Missing	9 (3.7)	8 (2.9)	17 (3.3)
WHO clinical stage			
Stage 1	86 (35.2)	56 (20.6)	142 (27.5)
Stage 2	55 (22.5)	97 (35.7)	152 (29.5)
Stage 3	103 (42.2)	119 (43.8)	222 (43.0)
ART regimen			
NNRTI-based	237 (97.1)	270 (99.3)	507 (98.3)
Dolutegravir-based	7 (2.9)	2 (0.7)	9 (1.7)
CD4 count in cells/μL, median (IQR)	527 (368-697)	572 (410-731)	552 (401-721)
Missing	69 (28.3)	73 (26.8)	151 (28.2)
Viral load			
≥1000 copies/mL	9 (3.7)	20 (7.4)	29 (5.6)
<1000 copies/mL	182 (74.6)	200 (73.5)	382 (74.0)
Missing	53 (21.7)	52 (19.1)	105 (20.3)
Self-reported adherence, mean (SD) %	98 (6)	98 (4)	98 (5)
Missing	0	1 (0.4)	1 (0.2)
Comprehensive ART knowledge			
No	41 (16.8)	35 (12.9)	76 (14.7)
Yes	203 (83.2)	237 (87.1)	440 (85.3)
HFIS score, median (IQR)	3 (0-8)	3 (0-7)	3 (0-7)
MOS-SS score, median (IQR)	26 (18-35)	32 (24-36)	30 (20-36)
AUDIT-C screening outcome			
Negative	232 (95.1)	251 (92.3)	483 (93.6)
Positive	8 (3.3)	19 (7.0)	27 (5.2)
Missing	4 (1.6)	2 (0.7)	6 (1.2)
AUDIT-C score, mean (SD)	0.3 (0.9)	0.5 (1.4)	0.4 (1.2)
Cost of transportation to facility (return), US $			
0	126 (51.6)	111 (40.8)	237 (45.9)
<2	34 (13.9)	14 (5.1)	48 (9.3)
2-5	52 (21.3)	67 (24.6)	119 (23.1)
>5	32 (13.1)	80 (29.4)	112 (21.7)

Abbreviations: ART, antiretroviral therapy; AUDIT-C, Alcohol Use Disorders Identification Test-Concise Test; HFIS, Household Food Insecurity Access Scale; MOS-SS, Medical Outcomes Study Social Support; NNRTI, nonnucleoside reverse transcriptase inhibitors; PHQ, Patient Health Questionnaire; SSQ, Shona Symptoms Questionnaire; WHO, World Health Organization.

^a^ The SSQ-14 score ranges from 0 (no symptom) to 14 (all symptoms present).

^b^ The PHQ-9 score is ranging from 0 (no depressive symptoms) to 27 (severe depressive symptoms).

To recruit this sample, we assessed the eligibility of 3706 individuals between October 5, 2018, and December 19, 2019, and excluded 3190 participants (86.1%) (Figure 1). Of the 595 participants who met inclusion criteria, 79 (13.3%) refused to participate or were lost to follow-up before the baseline visit. The last 12-month follow-up visit was on December 18, 2020.

### Outcomes

**Figure 2.**
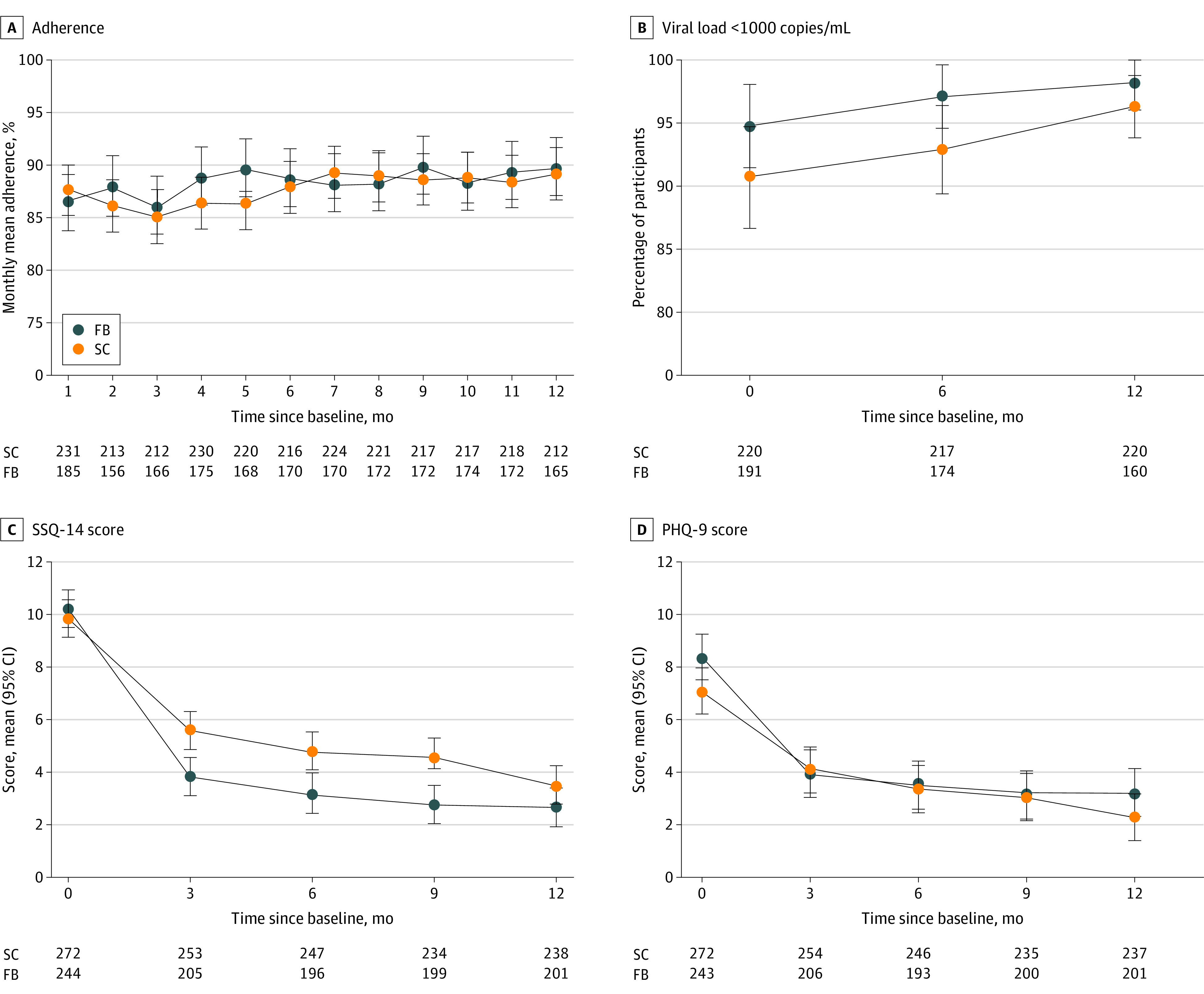
Adherence, Viral Load Suppression, Shona Symptoms Questionnaire (SSQ)-14 Score, and Patient Health Questionnaire (PHQ)-9 Score by Group Figure shows mean monthly adherence scores (A), proportions of participants with viral load of less than 1000 copies/mL (B), mean SSQ-14 scores, and mean PHQ-9 scores. Error bars represent 95% CIs for means and proportions. FB indicates Friendship Bench; SC, standard care.

In unadjusted analyses, the primary outcome of mean (SD) ART adherence between months 2 and 6 was slightly higher in the FB group (89.9% [18.4]) than in the SC group (87.2% [20.1]) (mean difference, 1.93 percentage points; 95% CI, −1.20 to 5.06 percentage points; *P* = .23), but there was little difference in mean ART adherence after month 6 (Figure 2A, Table 2). The intracluster coefficient of the primary outcome was 0.006. Overall, between months 1 and 12, mean ART adherence was similar (mean difference, 0.79 percentage points; 95% CI, −2.14 to 3.71 percentage points; *P* = .60) (Table 2). The odds of virologic suppression at 6 months (OR, 2.20; 95% CI, 0.79 to 6.14; *P* = .13) and 12 months (OR, 1.60; 95% CI, 0.42 to 6.05; *P* = .49) were higher in the FB than in the SC group, but differences failed to reach conventional levels of statistical significance (Figure 2B and Table 2). Declines in SSQ-14 scores from baseline to 3 months (difference, −1.65; 95% CI, −3.07 to −0.24), 6 months (difference, −1.57; 95% CI, −2.98 to −0.15), and 9 months (difference, −1.63; 95% CI, −3.05 to −0.22) were more pronounced in the FB than the SC group (Figure 2C and Table 2). There were no differences in the decline in the SSQ-14 scores from baseline to 12 months (Figure 2C and Table 2) and in PHQ-9 scores (Figure 2D and Table 2). There was little evidence for a difference in the odds of screening positively for common mental disorders (SSQ-14 >9) or depression (PHQ-9 >11) at 3, 6, 9, or 12 months (Table 2). Results were similar in the prespecified adjusted (Table 2) and post hoc sensitivity analyses for the intention to treat and per-protocol populations (eTable 1 and eTable 2 in Supplement 2).

**Table 2.** Effect of the Friendship Bench Intervention on Adherence, Viral Load, and Mental Health^a^

Outcome	Unadjusted analyses		Adjusted analyses^b^	
	Effect estimate	*P* value	Effect estimate	*P* value
Adherence, mean difference (95% CI)				
Month 2-6	1.93 (−1.20 to 5.06)	.23	1.79 (−1.71 to 5.29)	.32
Month 1-12	0.79 (−2.14 to 3.71)	.60	0.64 (−2.67 to 3.94)	.71
Viral suppression, odds ratio (95% CI)				
Month 6	2.20 (0.79 to 6.14)	.13	2.26 (0.79 to 6.45)	.13
Month 12	1.60 (0.42 to 6.05)	.49	1.75 (0.47 to 6.49)	.40
SSQ-14 score, mean difference (95% CI)				
Month 3	−1.65 (−3.07 to −0.24)	.02	−1.65 (−3.13 to −0.16)	.03
Month 6	−1.57 (−2.98 to −0.15)	.03	−1.56 (−3.05 to −0.07)	.04
Month 9	−1.63 (−3.05 to −0.22)	.02	−1.63 (−3.11 to −0.15)	.03
Month 12	−0.78 (−2.19 to 0.63)	.28	−0.77 (−2.25 to 0.71)	.31
PHQ-9 score, mean difference (95% CI)				
Month 3	−0.35 (−1.68 to 0.99)	.61	−0.49 (−1.82 to 0.85)	.47
Month 6	0.01 (−1.33 to 1.34)	.99	−0.14 (−1.47 to 1.20)	.84
Month 9	−0.04 (−1.39 to 1.30)	.95	−0.19 (−1.53 to 1.15)	.78
Month 12	0.74 (−0.60 to 2.08)	.28	0.60 (−0.74 to 1.93)	.38
SSQ-14 ≥9, odds ratio (95% CI)				
Month 3	0.38 (0.09 to 1.59)	.19	0.28 (0.07 to 1.12)	.07
Month 6	0.41 (0.09 to 1.83)	.25	0.30 (0.07 to 1.30)	.11
Month 9	0.34 (0.07 to 1.59)	.17	0.25 (0.05 to 1.13)	.07
Month 12	1.54 (0.33 to 7.15)	.58	1.12 (0.25 to 5.01)	.88
PHQ-9 ≥11, odds ratio (95% CI)				
Month 3	3.24 (0.60 to 17.46)	.17	2.16 (0.47 to 9.98)	.33
Month 6	3.17 (0.47 to 21.37)	.24	2.10 (0.35 to 12.47)	.41
Month 9	3.84 (0.49 to 30.30)	.20	2.55 (0.37 to 17.74)	.34
Month 12	3.11 (0.47 to 20.49)	.24	2.06 (0.36 to 11.85)	.42

Abbreviations: PHQ, Patient Health Questionnaire; SSQ, Shona Symptoms Questionnaire.

^a^ All analyses were prespecified and accounted for clustering of individuals in health facilities.

^b^ Adjusted for facility size, age, and sex.

### Retention

Retention in the FB and adherence interventions was high (Table 3). The median (IQR) number of sessions received among participants in the intervention group was 6 (6-6); 215 participants (88.1%) received all 6 sessions. In the intervention group, the proportion of participants who attended at least 1 group support session within a 3-month interval decreased from 165 (67.6%) to 125 (51.2%) from the first to the last interval. Few participants (6 [2.5%] in the FB group and 17 [6.3%] in the SC group) received antidepressants. Most participants with a viral load of 1000 copies/mL or more at the baseline visit (8 [72.7%] and 20 [76.9%] in FB and SC groups, respectively) attended all 3 adherence counseling sessions.

**Table 3.** Retention in Mental Health and Adherence Interventions

Intervention	Friendship bench (n = 244)	Standard of care (n = 272)	Total (n = 516)
Received allocated intervention	223 (91.4)	272 (100)	495 (95.9)
Friendship Bench intervention			
Individual counseling session 1 attended	235 (96.3)	NA	235 (45.5)
Individual counseling session 2 attended	230 (94.3)	NA	230 (44.6)
Individual counseling session 3 attended	229 (93.9)	NA	29 (44.4)
Individual counseling session 4 attended	224 (91.8)	NA	224 (43.4)
Individual counseling session 5 attended	219 (89.8)	NA	219 (42.4)
Individual counseling session 6 attended	215 (88.1)	NA	215 (41.7)
Group support attended			
Between month 0 and 3	165 (67.6)	NA	165 (32.0)
Between month 4 and 6	146 (59.8)	NA	146 (28.3)
Between month 7 and 9	141 (57.8)	NA	141 (27.3)
Between month 10 and 12	125 (51.2)	NA	125 (24.2)
Standard of care			
Nurse-led counseling attended	242 (99.2)	272 (100)	514 (99.6)
Antidepressant prescribed	6 (2.5)	17 (6.3)	23 (4.5)
**Enhanced adherence counseling**			
VL ≥1000 copies/mL at baseline visit			
Patients, No.	11	26	37
1-2 sessions attended	2 (18.2)	5 (19.2)	7 (18.9)
3 sessions attended	8 (72.7)	20 (76.9)	28 (75.7)
No session attended	1 (9.1)	0	1 (2.7)
Unknown	0	1 (3.8)	1 (2.7)
VL ≥1000 copies/mL 6-mo visit			
Patients, No.	6	17	23
1-2 sessions attended	0	5 (29.4)	5 (21.7)
3 sessions attended	5 (83.3)	10 (58.8)	15 (65.2)
No session attended	1 (16.7)	1 (5.9)	2 (8.7)
Unknown	0	1 (5.9)	1 (4.3)
VL ≥1000 copies/mL at 12-mo visit			
Patients, No.	3	9	12
1-2 sessions attended	0	2 (22.2)	2 (16.7)
3 sessions attended	0	2 (22.2)	2 (16.7)
Unknown	3 (100)	5 (55.6)	8 (66.7)

Abbreviations: NA, not applicable; VL, viral load.

### Safety Outcomes

In total, 16 participants reported self-harm or attempted self-harm; 11 (4.5%) were in the FB group and 5 (1.8%) were in the SC group. In the FB group, 9 participants (3.7%) reported a history of self-harm before baseline, and 2 participants (0.8%) reported a new incident self-harm event after baseline. No new incident self-harm event after baseline was reported in the SC group. No psychiatric hospitalizations occurred. Five participants died in the FB group and 1 in the SC group. All deaths were unrelated to study procedures, and no deaths by suicide were recorded or suspected (eTable 3 in Supplement 2).

## Discussion

In this cluster randomized trial, we examined the effect of the FB intervention on ART outcomes and mental health symptoms in adults with HIV in rural Zimbabwe. The intervention did not demonstrate a clear effect on ART adherence, viral suppression, and symptoms of depression (PHQ-9 scores) but had a beneficial effect on CMD symptoms (SSQ-14 scores). Characteristics of the study population at enrollment, including the high proportion of women, high baseline adherence, high baseline viral suppression rates, and relatively low mean PHQ-9 scores, may have contributed to the negative findings of this trial. The FB intervention was found to be a feasible and acceptable approach to address the mental health care needs of the rural study population. Implementing this psychological intervention led by lay health workers in rural health facilities was successful, with almost 90% of participants attending all 6 individual counseling sessions, reflecting their satisfaction and intervention appropriateness.

With the present study, 3 trials show that the FB intervention can reduce CMD symptoms (eFigure in Supplement 2). The first trial in urban Harare found that participants in the intervention group experienced a greater decrease in CMD and depression symptoms than participants in the enhanced standard care group.^28^ In contrast, our rural trial found a less pronounced decline in CMD symptoms and no effect on symptoms of depression. Differences could stem from urban vs rural settings, or the inclusion of a younger study population, or HIV-negative participants (42%) in the urban trial.^28^ Interestingly, decreases in the intervention group in CMD and depression symptoms were similar between the Harare trial and our study. The rural trial’s lower effectiveness can be attributed to differences in the rate of change within the control group. In the urban Harare trial,^28^ the 6-month scores remained high in controls, whereas in our rural trial, scores decreased both in the intervention and control group, suggesting that enhanced standard care may have been more effective in the rural setting. The third FB trial, involving Zimbabwean adolescents with HIV,^39^ showed improvements in CMD and depression symptoms compared with standard care, with relatively small differences in SSQ-14 and PHQ-9 scores of about 1 score point favoring the intervention.^39^

Two of the 3 trials examined virologic suppression (this study and the trial among adolescents^39^), and both failed to demonstrate any statistically significant effect (eFigure in Supplement 2). The present study also examined adherence and equally showed no robust intervention effect. A ceiling effect may have played a role in our study, as over 90% of participants had viral suppression and adherence was close to 90%. Given the well-established association between CMD and poor HIV outcomes,^9,10,11,12,13,14,15,16^ we expected lower baseline viral suppression and adherence rates. The large proportion of women in our study and the relatively older age may have contributed to these high rates. In a recent analysis of a South African cohort, we found considerably higher viral suppression and adherence rates in women than men and in older than in younger people.^40^ A ceiling effect is less likely for the trial among adolescents where more than one-third of participants had a viral load of 1000 copies/mL or more.^39^ Indeed, the nature of the FB intervention, which does not include skill-based adherence training, might be a more pervasive explanation for the lack of effect on viral suppression. Mental health interventions with integrated skill-based adherence training have been effective.^41^ For example, a trial of South African adults with depression showed that nurse-delivered cognitive-behavioral therapy improved depression scores, ART adherence, and viral suppression.^25^ Of note, a small pilot trial in Zimbabwe of a version of the FB intervention that incorporated skill-based training in adherence showed promising results regarding improved adherence and viral load suppression.^42^

### Strengths and Limitations

Our study’s strengths include a large sample size, long follow-up, and pragmatic study design resembling clinical practice conditions in rural Zimbabwe. Further strengths of our study include using electronic pill bottles for adherence monitoring and using a locally developed tool to measure CMD symptoms.^35^

Our study has several limitations. First, the mental health screening tools used in this study have been validated in Harare but not in the rural study setting, where our study took place.^35^ Second, men were underrepresented in our study (and the Harare study^28^), and it remains unclear how well the FB intervention works for them. Third, we could not evaluate the contribution of individual counseling, support groups, and nonspecific attention to the outcomes of the FB intervention. Additionally, although we did not reimburse for individual counseling or group support sessions, the $3 transport reimbursement for 3 monthly study visits might have influenced FB intervention retention, even though it was first paid after participants completed individual counseling.

## Conclusions

The FB intervention had no effect on adherence and viral suppression, possibly due to the absence of skill-based adherence training and ceiling effect. The intervention improved CMD symptoms, but the effect was smaller than previously shown in an urban setting. More work is needed to evaluate the effect of the approach on HIV outcomes in different populations, including young adults, men, and populations with more severe symptoms at higher risk of nonadherence and virologic failure. The further development of the FB intervention to incorporate adherence training may be a promising approach to reach those at high risk of poor HIV outcomes.

## Notes

Supplement 1.
Trial Protocol and Statistical Analysis Plan

Supplement 2. **eMethods. **Additional Information on Standard Care, Randomization, Adherence Measures, and Statistical Analyses **eTable 1. **Effect of the Friendship Bench Intervention on Adherence, Viral Load, and Mental Health: Results From Prespecified and Post Hoc Sensitivity Intention-to-Treat Analyses **eTable 2. **Effect of the Friendship Bench Intervention on Adherence, Viral Load, and Mental Health: Results From Prespecified and Post Hoc Sensitivity Per-Protocol Analyses **eTable 3. **Number of Participants With Safety-Relevant Outcomes by Trial Group **eFigure. **Forest Plot of Available Evidence of the Effect of the Friendship Bench Intervention on SSQ-14 Scores, PHQ-9 Scores, and Viral Suppression From 3 Trials

Supplement 3.
Nonauthor Collaborators

Supplement 4.
Data Sharing Statement
