# Supplementary material for: Effect of the Friendship Bench Intervention on Antiretroviral Therapy Outcomes and Mental Health Symptoms in Rural Zimbabwe: A Cluster Randomized Trial
Source: JAMA Netw Open. 2023 Jul 13;6(7):e2323205. doi: 10.1001/jamanetworkopen.2023.23205 (PMC10346120; doi:10.1001/jamanetworkopen.2023.23205)
Supplement: Supplement 1. — Trial Protocol and Statistical Analysis Plan [file jamanetwopen-e2323205-s001.pdf]

<< Effect of a psychological intervention on antiretroviral therapy outcomes and symptoms of common mental disorders in HIV-positive adults in rural Zimbabwe: cluster-randomized trial >>

Clinical Study Protocol

Study Type:	Clinical trial with investigational psychological intervention
Study Categorisation:	Risk category A (minimal risk) according to LHR
Study Registration:	The trial will be registered with clinicaltrials.gov and the Swiss National Clinical Trials Portal (SNCTP)
Study Identifier:	FB02

Sponsor

Prof. Dr. Matthias Egger
Institute of Social and Preventive Medicine
Finkenhubelweg 11, 3012 Bern, Switzerland
Email: matthias.egger@ispm.unibe.ch
Phone: +41 31 631 35 01

Principal Investigators:

Dr. Andreas Haas,
Institute of Social and Preventive Medicine
Finkenhubelweg 11, 3012 Bern, Switzerland
Email: andreas.haas@ispm.unibe.ch
Phone: +41 31 631 38 67

Cordelia Kunzekwenyika
SolidarMed Zimbabwe
33 Cary Street, Masvingo, Zimbabwe
Email: cordyku@gmail.com
Phone: +263 7 72525548

Protocol Version and Date: *Version 4, April 05, 2018*

Signature Page(s)

Study Identifier	FB02
Study Title	Effect of a psychological intervention on antiretroviral therapy outcomes and symptoms of common mental disorders in HIV-positive adults in rural Zimbabwe: cluster-randomized trial

The Sponsor, Principal Investigator and trial statistician have approved the protocol version 3 (dated 06.03.2018), and confirm hereby to conduct the study according to the protocol, current version of the World Medical Association Declaration of Helsinki, ICH-GCP guidelines or ISO 14155 norm if applicable and the local legally applicable requirements.

Sponsor: Prof. Dr. Matthias Egger

Bern, 5.4.2018 [signature]

Place/Date Signature

Principal-Investigator: Dr. Andreas Haas

Bern, 5/4/2018 Andreas Haas

Place/Date Signature

Signature Page(s)

Study Identifier	FB02
Study Title	Effect of a psychological intervention on antiretroviral therapy outcomes and symptoms of common mental disorders in HIV-positive adults in rural Zimbabwe: cluster-randomized trial

Local Principal Investigator at study sites in Zimbabwe

I have read and understood this trial protocol and agree to conduct the trial as set out in this study protocol, the current version of the World Medical Association Declaration of Helsinki, ICH-GCP guidelines or ISO 14155 norm and the local legally applicable requirements.

Site	Mashoko Hospital, Silveira Hospital, Nyika Rural Health Centre (RHC), Chikuku Rural Hospital (RH), Bikita RH, Pfupajena RHC, Marozva RHC, Mukore RHC, Murwira RHC, Negovano RHC, Gangare RHC, Ngorima RHC, Mutikizizi RHC, Hozvi RHC, Chirorwe RHC, Chitasa RHC
Principal investigator	Cordelia Kunzekwenyika

Masvingo, 11/04/2018 [signature]

Place/Date Signature

Table of Contents

STUDY SYNOPSIS **7**
STUDY SUMMARY IN GERMAN **10**
ABBREVIATIONS **11**
STUDY SCHEDULE **13**
1. STUDY ADMINISTRATIVE STRUCTURE **14**
1.1 Sponsor, Sponsor-Investigator 14
1.2 Principal Investigator(s) 14
1.3 Statistician ("Biostatistician") 14
1.4 Laboratory 14
1.5 Any other relevant Committee, Person, Organisation, Institution 15
2. ETHICAL AND REGULATORY ASPECTS **16**
2.1 Study registration 16
2.2 Categorisation of study 16
2.3 Competent Ethics Committee (CEC) 16
2.4 Ethical Conduct of the Study 16
2.5 Declaration of interest 16
2.6 Patient Information and Informed Consent 16
2.7 Participant privacy and confidentiality 17
2.8 Early termination of the study 17
2.9 Protocol amendments 17
3. BACKGROUND AND RATIONALE **18**
3.1 Background and Rationale 18
3.2 Investigational Treatment and Indication 19
3.3 Clinical Evidence to Date 20
3.4 Explanation for choice of comparator (or placebo) 20
3.5 Risks / Benefits 20
3.6 Justification of choice of study population 20
4. STUDY OBJECTIVES **22**
4.1 Overall Objective 22
4.2 Primary Objective 22
4.3 Secondary Objectives 22
4.4 Safety Objectives 22
5. STUDY OUTCOMES **23**
5.1 Primary Outcome 23
5.2 Secondary Outcomes 23
5.3 Other Outcomes of Interest 23
5.4 Safety Outcomes 23
6. STUDY DESIGN **24**
6.1 General study design and justification of design 24
6.2 Methods of minimising bias 24
6.2.1 Randomisation 24
6.2.2 Blinding procedures 24
6.2.3 Other methods of minimising bias 25
7. STUDY POPULATION **26**
7.1 Eligibility criteria 26

7.2 Recruitment and screening 26
7.3 Assignment to study groups 26
7.4 Criteria for withdrawal / discontinuation of participants 26
8. STUDY INTERVENTION 27
8.1 Identity of Investigational Intervention 27
8.1.1 Experimental Intervention: Problem-Solving Therapy 27
8.1.2 Control Intervention: Enhanced Standard of Care 27
8.1.3 Standard of Care for Patients on ART 27
8.2 Compliance with Study Intervention 27
8.3 Data Collection and Follow-up for Withdrawn Participants 27
8.4 Concomitant Interventions (treatments) 28
9. STUDY ASSESSMENTS 29
9.1 Study flow charts of study procedures and assessments 29
9.2 Assessments of outcomes 29
9.2.1 Assessment of primary outcome 29
9.2.2 Assessment of secondary outcomes 30
9.2.3 Assessment of eligibility criteria, baseline and other outcome measures 30
9.2.4 Assessment of safety outcomes 30
9.2.5 Assessments in participants who prematurely stop the study 30
9.2.6 Recruitment 30
9.2.7 Baseline assessment (visit 1) 30
9.2.8 Follow-up visits 30
9.2.9 Adherence measurement 31
9.2.10 Intervention group: problem-solving therapy and group support visits 31
9.2.11 Control group: enhanced standard of care visits 31
10. SAFETY 32
10.1 Definition and assessment of serious adverse events 32
10.2 Reporting of serious adverse events 32
11. STATISTICAL METHODS 33
11.1 Hypothesis 33
11.2 Determination of Sample Size 33
11.3 Planned Analyses 35
11.3.1 Datasets to be analysed, analysis populations 35
11.3.2 Primary Analysis 35
11.3.3 Secondary Analyses 35
11.3.4 Safety analysis 35
11.3.5 Deviation(s) from the original statistical plan 35
11.4 Handling of missing data and drop-outs 35
12. QUALITY ASSURANCE AND CONTROL 36
12.1 Data handling and record keeping / archiving 36
12.1.1 Case Report Forms 36
12.1.2 Specification of source documents 36
12.1.3 Record keeping / archiving 36
12.2 Data management 36
12.2.1 Data Management System 36
12.2.2 Data security, access and back-up 37

12.2.3 Analysis and archiving 37
12.2.4 Electronic and central data validation 37
12.3 Monitoring 37
12.4 Audits and Inspections 37
12.5 Confidentiality, Data Protection 37
12.6 Storage of biological material and related health data 37
13. PUBLICATION AND DISSEMINATION POLICY 38
14. FUNDING AND SUPPORT 39
14.1 Funding 39
15. INSURANCE 39
16. REFERENCES 40

STUDY SYNOPSIS

**Sponsor:**	Prof. Dr. Matthias Egger<br>Institute of Social and Preventive Medicine<br>Finkenhubelweg 11, 3012 Bern, Switzerland<br>Email: matthias.egger@ispm.unibe.ch<br>Phone: +41 31 631 35 01
**Principal Investigator:**	Dr. Andreas Haas,<br>Institute of Social and Preventive Medicine<br>Finkenhubelweg 11, 3012 Bern, Switzerland<br>Email: andreas.haas@ispm.unibe.ch<br>Phone: +41 31 631 38 67
**Local Principal Investigator:**	Cordelia Kunzekwenyika<br>SolidarMed Zimbabwe<br>33 Cary Street, Masvingo, Zimbabwe<br>Email: cordyku@gmail.com<br>Phone: +263 7 72525548
**Study Title:**	Effect of a psychological intervention on antiretroviral therapy outcomes and symptoms of common mental disorders in HIV-positive adults in rural Zimbabwe: cluster-randomized trial
**Short Title / Study ID:**	FB02
**Protocol Version and Date:**	Version 3 (dated 06.03.2018)
**Trial registration:**	The trial will be registered with clinicaltrials.gov and the Swiss National Clinical Trials Portal (SNCTP).
**Study category and Rationale**	Category A: The health-related intervention investigated entails only minimal risks and burdens
**Background and Rationale:**	Common mental disorders are highly prevalent among people living with HIV. Left untreated, common mental disorders cause substantial disability and undermine individuals' ability to adhere to antiretroviral therapy (ART), leading to poor HIV treatment outcomes. Management of mental disorders is a promising strategy to improve both mental health and HIV treatment outcomes among people on antiretroviral therapy who also suffer from common mental disorders. A recent cluster-randomized controlled trial from Harare, Zimbabwe showed that problem-solving therapy delivered by lay health workers effectively reduced symptoms of common mental disorders, but the effect of the intervention on HIV treatment outcomes and its effectiveness in the rural setting has not been studied. We aim to examine the effect of problem-solving therapy delivered by lay health workers on HIV treatment outcomes in rural Zimbabwe.
**Objective(s):**	Primary objective: To assess the effect of problem-solving therapy on HIV treatment outcomes.<br>Secondary objectives: To assess the effectiveness of problem-solving therapy on symptoms of common mental disorders among rural HIV-infected populations and to assess the prevalence of common-mental disorders among rural HIV-infected population on ART.
**Outcome(s):**	The primary outcome is the average difference in mean adherence between 2-6 months.<br>The secondary outcomes are average difference in mean adherence between 1-12 months, change from baseline in Shona Symptom Questionnaire (SSQ-14) and Patient Health Questionnaire (PHQ-9) score at 6 month and 12 month, change from baseline in proportion of participants with a viral load suppression (<20 copies per millilitre) at 6 months and 12 months.
**Study design:**	The study is designed as cluster-randomized, controlled, parallel two-arm multicentre trial with 1:1 allocation ratio.

**Inclusion / Exclusion criteria:**	Participants eligible for the trial must comply with all of the following criteria at screening:<br>1. Age ≥18 years<br>2. On first-line antiretroviral therapy for at least 6 months<br>3. Resident in Bikita District<br>4. Speak and understand English or Shona<br>5. Able to comprehend the information on the study<br>6. Screened positive for common mental disorders (SSQ-14 ≥9)<br>7. Provided written informed consent (or consent by thumb print) to participate study<br>Participants meeting any of the following criteria at screening will be excluded from the trial:<br>1. Current psychosis / cognitive impairment<br>2. Clinical AIDS (WHO clinical stage 4)<br>3. Known pregnancy or ≤3 months postpartum
**Measurements and procedures:**	Health facilities will be assigned in a 1:1 ratio to the intervention or the control group using a computer-generated, stratified blocked randomization. At each study facility, research assistants recruit eligible individuals in the waiting area of ART clinics and include individuals who provide written informed consent (or consent by thumb print). Study participants receive the psychological intervention or enhanced standard of care according to assignment of the health facility to intervention or control group. Outcomes will be assessed at baseline and at 3 monthly study visits.
**Study Product / Intervention:**	Participants in the intervention group receive individual counselling and a group activity to all services provided according to enhanced standard of care. Individual counselling is based on problem-solving therapy and delivered by lay health workers.
**Control Intervention (if applicable):**	Participants in the control group receive enhanced standard of care. In addition to all services provided according to national ART guidelines, participants receive information on and screening for common mental disorders, a nurse-led brief support counselling, assessment for antidepressant medication (fluoxetine) by the clinic nurse, and referral to a psychiatric facility if needed.
**Number of Participants with Rationale:**	We will recruit 30 participants at each of the 16 study facilities. In total, we will recruit 480 participants (240 in the intervention group and 240 in the control group).
**Study Duration:**	Ca. 18 months
**Study Schedule:**	February 2018 (or as soon as ethical approval is obtained)<br>August 2019 (anticipated duration of 18 months)
**Investigator(s):**	Dr. Dixon Chibanda<br>University of Zimbabwe<br>Department of Psychiatry<br>Prince Edward Street, Milton Park, Harare, Zimbabwe<br>Email: dichi@zol.co.zw<br>Phone: +263 712 204 107: 92<br><br>Dr. Janneke van Dijk<br>SolidarMed Zimbabwe<br>33 Cary Street, Masvingo, Zimbabwe<br>Email: J.vanDijk@solidarmed.ch<br>Phone: +263 77 6638747

**Study Centre(s):**	Mashoko Hospital, Silveira Hospital, Nyika Rural Health Centre (RHC), Chikuku Rural Hospital (RH), Bikita RH, Pfupajena RHC, Marozva RHC, Mukore RHC, Murwira RHC, Negovano RHC, Gangare RHC, Ngorima RHC, Mutikizizi RHC, Hozvi RHC, Chirorwe RHC, Chitasa RHC
**Statistical Considerations:**	We will use linear and logistic mixed effect models and to assess differences in study outcomes. Primary analysis will be by intention to treat. The study was powered to detect a moderate effect size in the primary outcome with a chance of 90%.
**GCP Statement:**	This study will be conducted in compliance with the protocol, the current version of the Declaration of Helsinki, the ICH-GCP or ISO EN 14155 (as far as applicable) as well as all national legal and regulatory requirements.

STUDY SUMMARY IN GERMAN

**Titel:**	Studie zur Wirksamkeit einer psychologischen Intervention zur Verbesserung des Behandlungserfolgs von antiretroviraler Therapie sowie zur Linderung der Symptomatik häufiger psychischer Erkrankungen bei HIV-infizierten Erwachsenen im ländlichen Zimbabwe
**Untersuchte Krankheiten:**	Humane Immundefizienz-Virus (HIV), häufige psychische Erkrankungen (z.B. Depression und generalisierte Angststörung)
**Zusammenfassung**	Diese Studie untersucht die Wirksamkeit einer psychologischen Intervention zur Verbesserung des Behandlungserfolgs von antiretroviraler Therapie sowie zur Linderung der Symptomatik häufiger psychischer Erkrankungen bei HIV-infizierten Erwachsenen. Dafür werden Patienten in 16 Gesundheitszentren in ländlichen Gebieten in Zimbabwe rekrutiert. Die psychologische Intervention wird in der Hälfte der Gesundheitszentren eingeführt. Die übrigen Gesundheitszentren dienen als Kontrollgruppe. Die Zuweisung der Gesundheitszentren zur Interventions- beziehungsweise Kontrollgruppe, erfolgt zufällig. Der primäre Endpunkt, zur Messung der Wirksamkeit der psychologischen Intervention, ist der durchschnittliche Unterschied in den Mittelwerten der Adhärenz über die Monate 2-6 zwischen den beiden Gruppen. Sekundäre Endpunkte sind der durchschnittliche Unterschied in den Mittelwerten der Adhärenz über die Monate 1-12, der durchschnittliche Unterschied in den Mittelwerten der Veränderung des Shona Symptom Questionnaire (SSQ-14) Wertes und des Patient Health Questionnaire (PHQ-9) Wertes nach 6 beziehungsweise 12 Monaten und die Veränderung des Anteils der Studienteilnehmer*innen mit einer HIV Viruslast von unter 20 Kopien pro Milliliter Blut zwischen den beiden Gruppen.
**Untersuchte Intervention:**	Studienteilnehmer*innen in der Interventionsgruppe nehmen an sechs Einzelsitzungen Gesprächstherapie und sechs Sitzungen Gruppentherapie teil. Die Einzelsitzungen werden von einer angelernten Mitarbeiterin ohne formale Qualifikation im Gesundheitswesen durchgeführt. Eine Sitzung dauern circa 30-45 Minuten. Sitzungen finden auf dem Gelände der Gesundheitszentren statt. Die Therapiesitzungen basieren auf dem Ansatz der Problemlösungstherapie. Ziel der Therapie ist, Alltagsprobleme, die zu schlechter psychischer Gesundheit führen, zu erkennen und Lösungsansätze zur Verbesserung der Gesundheit zu erarbeiten. Studienteilnehmer*innen werden dabei unterstützt, Probleme die zu schlechter Adhärenz führen, zu erkennen und Strategien zur Verbesserung der Adhärenz zu entwickeln. Nach vier Sitzungen Einzeltherapie werden die Studienteilnehmer*innen der Interventionsgruppe dazu eingeladen an Gruppentherapiesitzungen teilzunehmen, in denen sie sich mit anderen Studienteilnehmer*innen austauschen können und unter Anleitung eine handwerkliche Fahrigkeit (z.B. das Knöpfen von Bastkörben) erlernen.
**Einschlusskriterien:**	Studienteilnehmer*innen müssen die folgenden Einschlusskriterien erfüllen:<br>1. Mindestalter 18 Jahre;<br>2. seit mindestens 6 Monaten auf antiretroviraler Therapie;<br>3. SSQ-14 Score von mindestens 9 Punkten;<br>4. Wohnort im Bikita District;<br>5. ausreichend Sprachkenntnisse in Shona oder im Englischen;<br>6. ausreichend kognitive Fähigkeiten um die Informationen der Studie zu begreifen;<br>7. schriftliche Einverständniserklärung zur Teilnahme an der Studie.<br>Personen die eines der folgenden Kriterien erfüllen, sind von der Teilnahme an der Studie ausgeschlossen:<br>1. akute Psychose oder kognitive Beeinträchtigung;<br>*2.* weit fortgeschrittene HIV Infektion (Stufe 4 nach der Klassifikation der Weltgesundheitsorganisation);<br>3. gegenwärtige Schwangerschaft oder Geburt eines Kindes innerhalb der letzten 3 Monate.

ABBREVIATIONS

AE	Adverse Event
ART	Antiretroviral therapy
CA	Competent Authority (e.g. Swissmedic)
CEC	Competent Ethics Committee
CRF	Case Report Form
ClinO	Ordinance on Clinical Trials in Human Research *(in German: KlinV, in French: OClin)*
CMD	Common mental disorders
eCRF	Electronic Case Report Form
CTCAE	Common terminology criteria for adverse events
DSUR	Development safety update report
EDC	Electronic data capture
GCP	Good Clinical Practice
IB	Investigator's Brochure
Ho	Null hypothesis
H1	Alternative hypothesis
HFG	Humanforschungsgesetz (Law on human research)
HMG	Heilmittelgesetz
HRA	Federal Act on Research involving Human Beings
IMP	Investigational Medicinal Product
IIT	Investigator-initiated Trial
ISO	International Organisation for Standardisation
ITT	Intention to treat
KlinV	Verordnung über klinische Versuche in der Humanforschung *(in English: ClinO, in French OClin)*
LPTh	Loi sur les produits thérapeutiques
LRH	Loi fédérale relative à la recherche sur l'être humain
MD	Medical Device
MEMS	Medication Event Monitoring System
MRCZ	Medical Research Council of Zimbabwe
OClin	Ordonnance sur les essais cliniques dans le cadre de la recherche sur l'être humain *(in German : KlinV, in English : ClinO)*
PHQ-9	Patient Health Questionnaire
PI	Principal Investigator
SDV	Source Data Verification
SOP	Standard Operating Procedure
SPC	Summary of product characteristics
SSQ-14	Shona Symptoms Questionnaire
SUSAR	Suspected Unexpected Serious Adverse Reaction
TMF	Trial Master File

STUDY SCHEDULE

Figure 1: Gantt-chart of showing facility timeline of staggered implementation of intervention and control arm at study facilities.

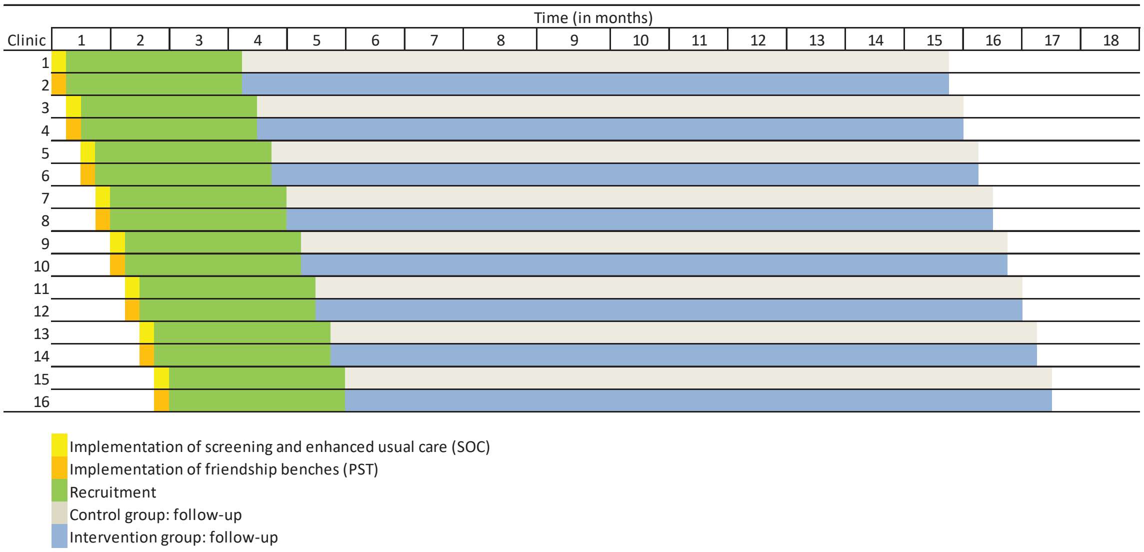

Figure 2: Gantt-chart of showing participant timeline of counselling sessions and outcome assessments.

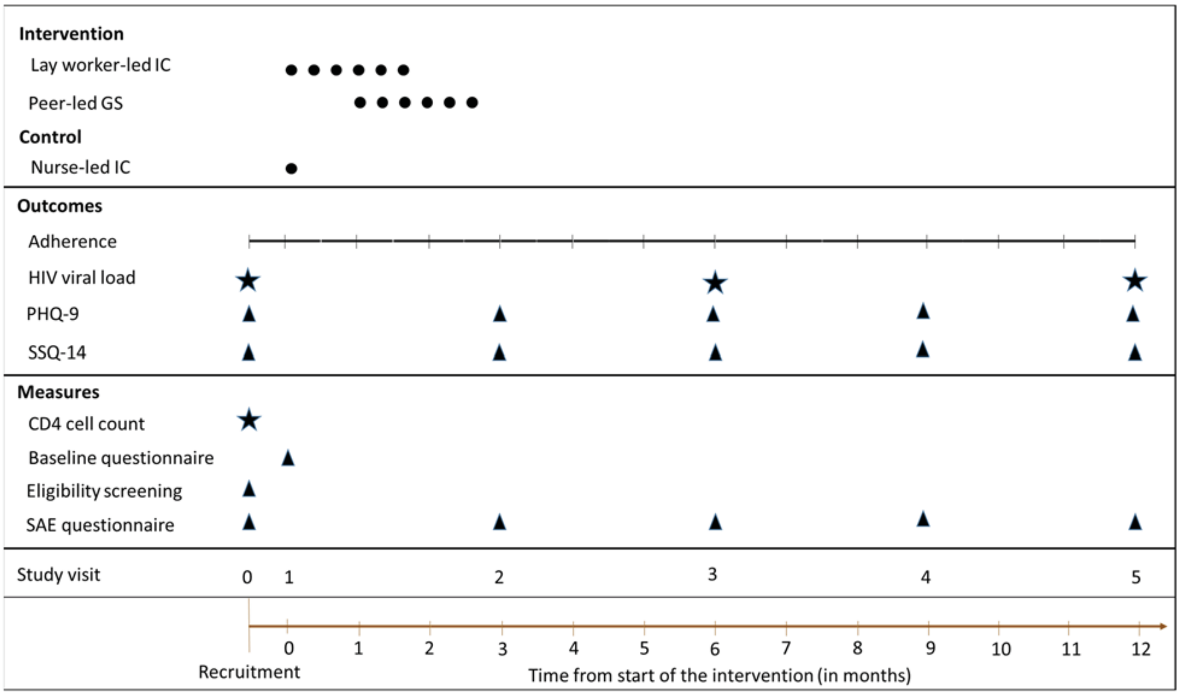

PST: Problem-solving therapy; MEMS: Medication Event Monitoring System; PHQ-9: Patient Health Questionnaire; SSQ-14: Shona Symptoms Questionnaire; SAE: Serious Adverse Event.

1. STUDY ADMINISTRATIVE STRUCTURE

Prof. Matthias Egger acquired funding for the study and has the overall responsibility for the initiation and management of the trial. Dr. Andreas Haas oversees research design, monitoring, and data management and is responsible for project management and reporting in Switzerland. Ms. Kunzekwenyika overseas implementation of fieldwork and data collection, on-site monitoring, training of research assistants and is responsible for project management and reporting in Zimbabwe. Dr. Chibanda from the Department of Psychiatry at University of Zimbabwe developed the friendship bench intervention overseas implementation of the friendship bench program and training and supervision of lay health workers. Dr. Trelle, Dr. Limacher and Ms. Hossmann from the Clinical Trials Unit, University of Bern, oversea central data monitoring and advise on research design, data management, statistical analysis, research ethics, good clinical practice, and monitoring.

1.1 Sponsor, Sponsor-Investigator

Prof. Dr. Matthias Egger

University of Bern

Institute of Social and Preventive Medicine (ISPM)

Finkenhubelweg 11, 3012 Bern, Switzerland

Email: matthias.egger@ispm.unibe.ch

Phone: +41 31 631 35 01

Prof. Egger acquired funding for the study and has the overall responsibility for the initiation and management of the trial. He contributed to the design of the study, and he will contribute to interpretation of data, and writing of reports.

1.2 Principal Investigator(s)

Dr. Andreas Haas,

University of Bern

Institute of Social and Preventive Medicine (ISPM)

Finkenhubelweg 11, 3012 Bern, Switzerland

Email: andreas.haas@ispm.unibe.ch

Phone: +41 31 631 38 67

Cordelia Kunzekwenyika

SolidarMed Zimbabwe

33 Cary Street, Masvingo, Zimbabwe

Email: cordyku@gmail.com

Phone: +263 7 72525548

1.3 Statistician ("Biostatistician")

Dr. Andreas Limacher

University of Bern

Clinical Trials Unit (CTU)

Finkenhubelweg 11, 3012 Bern, Switzerland

Email: andreas.limacher@ctu.unibe.ch

Phone: +41 31 631 35 10

1.4 Laboratory

Masvingo provincial laboratory

Masvingo Provincial Hospital

75 Hay Robertson Street, Masvingo, Zimbabwe

Silveira district laboratory
Silveira District Hospital
Nyika, Zimbabwe

1.5 Any other relevant Committee, Person, Organisation, Institution

Dr. Dixon Chibanda
University of Zimbabwe
Department of Psychiatry
Tel: + 263 7 12204107
Email: dichi@zol.co.zw
Dr. Chibanda overseas training and supervision of lay health workers and implementation of the friendship bench intervention.

Dr. Janneke van Dijk
SolidarMed Zimbabwe
33 Cary Street, Masvingo, Zimbabwe
Email: J.vanDijk@solidarmed.ch
Phone: +263 77 6638747

Dr. van Dijk is the country director of SolidarMed Zimbabwe.

Dr. Sven Trelle
University of Bern
Clinical Trials Unit (CTU)
Finkenhubelweg 11, 3012 Bern, Switzerland
Email: sven.trelle@ctu.unibe.ch
Phone: +41 31 631 35 04

Dr. Trelle is the co-director of CTU Bern.

Hossmann Stefanie
University of Bern
Clinical Trials Unit (CTU)
Finkenhubelweg 11, 3012 Bern, Switzerland
E-Mail: stefanie.hossmann@ctu.unibe
Phone: +41 31 631 30 87

Stefanie Hossmann is Clinical Trial Manager at CTU Bern.

2. ETHICAL AND REGULATORY ASPECTS

The decision of the CEC and foreign competent authority concerning the conduct of the study will be made in writing to the Sponsor-Investigator before commencement of this study. The clinical study can only begin once approval from all required authorities has been received. Any additional requirements imposed by the authorities shall be implemented.

2.1 Study registration

The trial will be registered with clinicaltrials.gov and the Swiss National Clinical Trials Portal (SNCTP).

2.2 Categorisation of study

Category A: An "other clinical trial" where the health-related intervention investigated entails only minimal risks and burdens.

2.3 Competent Ethics Committee (CEC)

The principal investigators ensure that approval from an appropriately constituted Competent Ethics Committee (CEC) will be sought for the clinical study and that all reporting duties will be fulfilled within the allowed periods.

2.4 Ethical Conduct of the Study

The study will be carried out in accordance to the protocol and with principles enunciated in the current version of the Declaration of Helsinki, the guidelines of Good Clinical Practice (GCP) issued by ICH, in case of medical device: the European Directive on medical devices 93/42/EEC and the ISO Norm 14155 and ISO 14971, the Swiss Law and Swiss regulatory authority's requirements. The CEC will receive annual safety and interim reports and be informed about study stop/end in agreement with local requirements.

2.5 Declaration of interest

Sponsor, principal and co-investigators declare that they have no competing interests with the conduct of this study.

2.6 Patient Information and Informed Consent

The investigators will explain to each participant the nature of the study, its purpose, the procedures involved, the expected duration, the potential risks and benefits and any discomfort it may entail. The investigators will obtain verbal consent to screen for common mental disorders and to extract data from medical records to assess eligibility of persons (age, current WHO clinical stage, ART start date, ART regiment). Research assistants will extract data of participants who have consented verbally and administer a brief screening questionnaire (Shona Symptom Questionnaire [SSQ-14]). Individual with a SSQ-14 score of 9 or higher who meet all other eligibility criteria will be included in the study if they are willing to participate. Participants who are not eligible for the study will be asked to provide verbal consent that their screening and questionnaire data may be used for deidentified statistical analysis to assess the prevalence of common mental disorders common-mental disorders among people on antiretroviral therapy. Each eligible participant will be informed that the participation in the study is voluntary and that he/she may withdraw from the study at any time and that withdrawal of consent will not affect his/her subsequent medical assistance and treatment. The participant will be informed that his/her medical records may be examined by authorised individuals other than their treating physician. All participants for the study will be provided a participant information sheet and a consent form describing the study and providing sufficient information for participant to make an informed decision about their participation in the study. After potential study participants received all relevant information they will be given time to decide whether to participate or not. During this time, potential participants will be offered refreshments and are given the opportunity to go for a short walk. Individuals will be given more time to make their decision if needed. The patient information sheet and the consent form will be submitted to the CEC and to the Medical Research Council of Zimbabwe (MRCZ) to be reviewed and approved. The formal consent of a participant, using the approved consent form, must be obtained before the participant is submitted to any study procedure. The participant should read and consider the statement before signing and dating the informed consent form, and should be given a copy of the signed document. If a person is unable to read an impartial witness should be present during the entire informed consent discussion. After the written informed consent form, is read and explained to the

participant and after the participant has consented by thumbprint to the participation in the trial, the witness should sign and personally date the consent form. By signing the consent form, the witness attests that the information in the consent form and any other written information was accurately explained to, and apparently understood by, the participant, and that informed consent was freely given by the participant. The consent form must also be signed and dated by the investigator (or his designee) and it will be retained as part of the study records.

2.7 Participant privacy and confidentiality

The investigator affirms and upholds the principle of the participant's right to privacy and that they shall comply with applicable privacy laws. Especially, anonymity of the participants shall be guaranteed when presenting the data at scientific meetings or publishing them in scientific journals.

Individual subject medical information obtained as a result of this study is considered confidential and disclosure to third parties is prohibited. Subject confidentiality will be further ensured by utilising subject identification code numbers to correspond to treatment data in the computer files.

For data verification purposes, authorised representatives of the Sponsor (-Investigator), a competent authority (e.g. Swissmedic), or an ethics committee may require direct access to parts of the medical records relevant to the study, including participants' medical history.

2.8 Early termination of the study

The Sponsor-Investigator may terminate the study prematurely according to certain circumstances, for example:

- ethical concerns,
- insufficient participant recruitment,
- when the safety of the participants is doubtful or at risk, respectively,
- alterations in accepted clinical practice that make the continuation of a clinical trial unwise,
- early evidence of benefit or harm of the experimental intervention

2.9 Protocol amendments

Substantial amendments are only implemented after approval of the CEC and CA respectively.

Under emergency circumstances, deviations from the protocol to protect the rights, safety and well-being of human subjects may proceed without prior approval of the sponsor and the CEC/CA. Such deviations shall be documented and reported to the sponsor and the CEC/CA as soon as possible.

All Non-substantial amendments are communicated to the CA as soon as possible if applicable and to the CEC within the Annual Safety Report (ASR).

3. BACKGROUND AND RATIONALE

3.1 Background and Rationale

Common mental disorders (CMDs) are highly prevalent among people living with HIV. In Zimbabwe, about 15% of the adult population are infected with HIV and about two thirds of the people living with HIV show symptoms of CMDs.[1,2]

CMDs undermine individuals' ability to adhere to HIV treatment. In Zimbabwe, all people with confirmed HIV diagnosis are eligible for antiretroviral therapy (ART).[3] Patients on ART need to take antiretroviral medication every day and exactly as prescribed. Strict medication adherence is crucial for the long-term effectiveness of ART, including sustained viral load suppression, immune recovery, reduced risk of drug resistance, decreased risk of opportunistic infections, survival and prevention of HIV transmission.[4–9] CMDs have consistently been shown to be associated with poor HIV treatment outcomes including poor adherence to ART, loss to follow-up, lack of virological suppression, and faster progression to advanced stages of disease.[10–14]

Interventions to address low adherence to ART among HIV-positive people with CMDs are needed to improve their long-term prognosis. Management of mental and substance use disorders is a promising strategy to improve both mental health and HIV treatment outcomes in this population. We systematically searched PubMed for randomized clinical trials, which assessed the effectiveness of psychological or pharmacological treatment of CMDs on mental health and HIV treatment outcomes in adults. We identified seven relevant studies.

In three studies, the interventions significantly improved mental health outcomes, but had no effect on ART adherence and viral load suppression. Pyne and colleagues tested the effect of collaborative care model among HIV+ adults with depressive symptomology in the United States (US). Participants in the intervention group responded well to depression treatment and were more than twice as likely to be in depression remission as participants in the control group. However the intervention had no effect on self-reported 4 days recall adherence to antiretroviral medication.[15] Pence and colleagues assessed the effect of antidepressant treatment on HIV and depression outcomes among HIV+ adults with major depressive disorder in the US. The intervention had an effect on depression severity, depression remission, and suicidal ideation, but no effect on 12 months unannounced telephone-based pill count adherence or HIV viral load.[16] Tsai et al. evaluated the effect of directly observed antidepressant treatment among HIV-positive homeless adults with depression in the US. Antidepressant medication reduced depression symptom severity, increased response and remission, but had no effect on HIV viral load and ART adherence assessed by unannounced pill count.[17]

In four studies, the interventions significantly improved mental health and adherence outcomes. In a study by Safren and colleagues, cognitive behavioral therapy with adherence counseling had a significant treatment effect on adherence and depression among HIV-positive people with depression and a history of intravenous drug use in the US. Improvements in depression were maintained after discontinuation of depression treatment, but gains in adherence were not.[18] In two studies, Safran and colleagues assessed the effect of cognitive behavioral therapy for depression with adherence counselling among HIV+ adults with depression in the US. The interventions effectively reduced depression and adherence measured using electronic pill bottles. Changes in depression and adherence were maintained over 12 months follow-up period.[19,20] These findings were confirmed in a study by Simoni and colleagues who showed that cognitive-behavioral therapy for adherence and depression with alarmed pillbox had a significant effect on depression and adherence among HIV-positive Latinos with imperfect adherence and depressive symptomatology in the US–Mexico border region. Effects on adherence and depression were maintained over 12 months on follow-up.[21] Despite improvements in adherence, none of the studies showed a significant effect on virological outcomes.[18–21]

Management of mental and substance use disorders is a promising strategy to improve both mental health and HIV treatment outcomes, but evidence on the effect of mental health interventions on HIV treatment outcomes is inconsistent, with no body of evidence from Africa. A recent cluster-randomized controlled trial from Harare, Zimbabwe showed that problem-solving therapy delivered by lay health workers effectively reduced symptoms of CMDs, but the effect of the intervention on HIV treatment outcomes and its effectiveness in the rural setting has not been studied.[22] We aim to examine the effect of problem-solving therapy delivered by lay health workers on HIV treatment outcomes in rural Zimbabwe.

3.2 Investigational Treatment and Indication

We investigate the effectiveness of a brief psychological intervention for the management of CMDs. The indication for the intervention is a positive screening for CMDs defined as SSQ-14 score ≥9. The SSQ-14 is a locally developed screening tool for CMDs.[23] SSQ-14 score ≥9 is an inclusion criterion of our study. All study participants assigned to the intervention group receive the psychological intervention. The intervention consists of individual counselling and a group activity in addition to all services provided according to enhanced standard of care. Individual counselling is based on problem-solving therapy. Lay health workers deliver therapy sessions. Three lay health workers will be trained at each facility. Therapy sessions take place in a private area of the health facilities, which can be an outside area. Participants receive six weekly counselling sessions of 30-45 minutes over a six-week period. Therapy takes a manualized structured approach to identify practical problems and teaches a positive attitude towards resolving them. Participants will be actively encouraged to identify and tackle problems leading to suboptimal adherence to ART. Table 1 gives an overview of the intervention. More details on the intervention have been described in detail elsewhere.[22,24] After four session of individual counselling, participants are invited to join a peer-led group activity. In the group activity, participants are taught an income generating skill (e.g. to produce bags from recycled plastic) and have the opportunity to share personal experience with former participants of the interventoin.[22,24] Participants in the intervention group are also offered enhanced standard of care.

Table 1: Description of the problem solving therapy intervention

Theoretical basis	Cognitive behavioral therapy
Delivering agent	Lay health workers (Health promoters). About 55 years old, female, about 8 years of education, previous training in health-related subjects (e.g. home based care for people living with HIV & AIDS, community follow-up of persons on HIV/TB treatment, or delivering community health education and promotion).
Structure of intervention	Six weekly sessions of 30–45 min delivered through lay health workers over 6 weeks, including home visits where deemed necessary. The first session lasting between 45 and 60 min
Structure of sessions and areas covered	Part 1. Problem identification (Kuvhura pfungwa): (A) Share Shona Symptom Questionnaire information with client, explain symptoms in relation to kufungisisa (kufungisisa is a Shona idiom for non-psychotic mental illness that means "thinking too much"25) (B) Actively listen to clients story, identify and list problems raised, clearly define problem/s. Problem exploration (kusimudzira): (C) understand the story, help client prioritise problems by summarizing and asking if you have missed anything, (D) brainstorm practical/feasible solutions, outline the options available (these have to come from client), encourage client to think over solutions of each problem before having the client decide which one to focus on. Help client to come up with a specific, measurable, achievable and realistic solution (don't tell client what to do). Agree what the client will do before you next meet and set appointment date.<br>Part 2. Reassure (Kusimbisa) (E) Home visit if needed before second meeting, otherwise see again on the bench, how did it go? Went well, then reassure praise encourage. If no progress or new obstacles present then go back to Part 1 contents, redefine problem and goals, what were the obstacles? Problem solve around obstacles and give homework again and reassure, you can phone or send SMS to reassure client (up to 6 per client).<br>Part 3 (kusimbisisa). (F) Summarize session 1, how did it go? Going well then reassure and encourage. Still having problems with agreed plan? Go back to Part 1 again or if you feel frustrated go to supervisor.
Remember	Action plan: (G) Zero in on a specific solution, focus on what client wants to do and not what you think should be done, (H) How, when, what assistance is needed? Referral if necessary (I). Identify activities the person used to find rewarding and which matter to them and encourage these (J), Implementation: (K) How will it be done? Motivate; homework, Refer after 4th session to support group. Follow up: (L) What has been achieved? What were/are the obstacles if any? Go back to Part 1 as often as needed during the 6 sessions (M) Reinforce. What has been achieved, repeat SSQ score. (N) No improvement refer to supervisor. nurse counsellor

Tools	SSQ-14, Friendship bench manual, referral protocol
Supervisor	Weekly group supervision by a clinician (Psychologist) or senior study team member trained in PST. Access to direct mobile voice call to support team.

Adopted from Chibanda et al. JAMA 2016.[22]

3.3 Clinical Evidence to Date

The intervention has successfully been tested in an urban general adult population in Zimbabwe. A recent cluster-randomized controlled trial from Harare showed that the intervention could be safely delivered by lay health workers and effectively reduced symptoms of CMDs.[22] However, the effect of the intervention on HIV treatment outcomes and its effectiveness in the rural setting and on HIV-infected populations has not been studied.[22] Some studies from the United States showed that cognitive behavioral interventions can have a positive effect on ART adherence.[18–21] In other studies from the United States countries psychological or pharmacological treatment of depression improved mental health, but not HIV treatment outcomes.[15–17] To the best of our knowledge, there are no data on the effect of management of CMDs on HIV treatment outcomes from low- and middle income settings.

3.4 Explanation for choice of comparator (or placebo)

We compare the effect of problem-solving therapy with enhanced standard of care for CMDs. Participants from the control group receive information on available routine services for CMD, a nurse-led brief support counselling, and undergo an assessment for antidepressant medication (fluoxetine) by the clinic nurse. Participants with severe mental illnesses, which cannot be managed in study facilities, will be referred to psychiatric facilities.

3.5 Risks / Benefits

Research participants are at minimal risk of breach of confidentiality. To avoid breach of confidentiality all data will be kept strictly confidential and personal information that can be used to identify participants will be kept strictly confidential separately from other study data and can only be accessed by the research coordinator (to be named) and the local PI (Ms. Kunzekwenyika). All data will be stored on encrypted hard-drives. For more details, see section 12.5.

Research participants and their communities will benefit from the study. CMDs cause a great burden of disease and are associated with poor HIV treatment outcomes. Currently, study sites do not systematically screen for CMDs and have very limited human resource capacity to manage mental illness. Therefore, CMDs are rarely diagnosed or treated at the study setting and participants. The study will substantially improve diagnosis and management of CMDs at all study sites. All study participants will benefit from enhanced standard of care that will be implemented at all study sites. Enhanced standard of care consists of screening and management of CMDs, a nurse-led brief support counselling, assessment for antidepressant medication by the clinic nurse, and if needed, referral to a psychiatric facility. Furthermore, all study participants will benefit electronic adherence monitoring and viral load testing that will be done in addition to testing during usual care. Participants in the intervention group may also benefit from the friendship bench intervention. If the intervention will be found to be effective, we will implement the intervention in all study facilities at the end of our study and all study patients will be offered to receive the friendship bench intervention. In addition to direct benefits to study participants, our study is likely to have further benefits to the community of study participants. We will train at least 24 lay health workers in problem-solving therapy and therefore substantially increase the human resource capacity for the management of CMDs in the study settings. Lastly, our study will generate evidence on the effectiveness of the friendship bench intervention in the rural setting and will evaluate whether friendship benches are an evidence-based intervention to improve HIV treatment outcomes in people with CMDs. In conclusion, we believe that it is well justified to conduct our study because the direct benefits to study participants and anticipated benefits to the community outweigh the minimal risk to study participants.

3.6 Justification of choice of study population

CMDs are highly prevalent among people living with HIV. In Zimbabwe, about 15% of the adult population are infected with HIV and about two thirds of the people living with HIV show symptoms of CMDs.[1,2] CMDs undermine individuals' ability to adhere to HIV treatment and are associated with poor HIV treatment outcomes including loss to follow-up, lack of virological suppression, and faster progression of disease.[10–14] Interventions to address low adherence to ART among HIV-positive people

with mental disorders are needed to improve their long-term prognosis. Management of mental disorders is a promising strategy to improve both mental health and HIV treatment outcomes in this population. However, in rural low-income settings CMDs are rarely diagnosed or treated due to a large shortage of mental health professionals. Delivery of psychological interventions by lay health workers is a feasible approach to improve the mental health in these settings. A recent cluster-randomized controlled trial from Zimbabwe showed that psychological interventions can safely and effectively delivered by lay health workers, but this approach has not been tested in rural HIV-infected populations and it is unclear whether improved mental health outcomes also lead to better ART adherence.[22] We aim to investigate the effect of problem-solving therapy delivered by lay health workers on mental health and HIV treatment outcomes in rural HIV-infected populations in Zimbabwe. Our study does not include vulnerable populations.

4. STUDY OBJECTIVES

4.1 Overall Objective

This study aims to compare the effectiveness of problem-solving therapy for CMDs against enhanced standard of care among rural HIV-infected population in Zimbabwe.

4.2 Primary Objective

To assess the effect of problem-solving therapy on HIV treatment outcomes.

4.3 Secondary Objectives

To assess the effectiveness of problem-solving therapy on symptoms of CMDs among rural HIV-infected populations and to estimate the prevalence of common-mental disorders among rural HIV-infected population on antiretroviral therapy.

4.4 Safety Objectives

To assess the safety of the intervention in terms of incidence of the serious adverse events (SAE) 1) psychiatric hospitalization 2) suicide attempt, and 3) suicide (see 5.4).

5. STUDY OUTCOMES

5.1 Primary Outcome

The primary outcome is the average difference in mean adherence between 2-6 months between the groups. We will calculate participants' 4-weekly mean adherence scores as the percentage of doses taken per interval. For regimens that have to be taken once daily, a dose was considered as taken if the participant opens the electronic pill bottle within -8 to +16 hours of the designated dosing time. For regimens that have to be taken twice daily, a dose was considered as taken if the participant opens the electronic pill bottle within -4 to +8 hours of the designated dosing time for patients.

5.2 Secondary Outcomes

Secondary outcomes are average difference in mean adherence between 1-12 months, change from baseline in SSQ-14 and PHQ-9 score at 6 month and 12 month, change from baseline in proportion of participants with a viral load suppression (<20 copies per milliliter) at 6 months and 12 months.

5.3 Other Outcomes of Interest

Other relevant outcomes are the prevalence of common-mental disorders (SSQ-14 ≥9) and depression (PHQ-9 ≥11) among rural HIV-infected population on ART.

5.4 Safety Outcomes

Safety outcomes are difference in the incidence of 1) psychiatric hospitalization 2) suicide attempt, and 3) suicide up to 12 months.

6. STUDY DESIGN

6.1 General study design and justification of design

The study is designed as cluster-randomized, controlled, parallel two-arm multicenter, open-label superiority trial with 1:1 allocation ratio to compare the effectiveness of problem-solving therapy against enhanced standard of care on mental health and HIV treatment outcomes among HIV-infected adults on ART who screen positive for CMD. A randomized controlled trial is the most rigorous designs to evaluate the effectiveness of interventions. To prevent contamination between study arms and to avoid creating inequalities between individuals receiving HIV care at the same facility randomization of health facilities (clusters) was chosen. We will include 480 participants. The intervention will be compared against enhanced stand of care for CMDs among HIV-infected adults. We will follow study participants for 12 months. We aim to implement the study in 16 of the larger health facilities in Bikita District in rural Zimbabwe (Table 2). All study facilities are supported by the non-governmental organization SolidarMed.[26,27]

Table 1: Characteristics of study facilities

	Facility	Type of clinic	Active ART (N)
1	Mashoko	Hospital	1'186
2	Silveira	Hospital	1'023
3	Nyika	Rural Health Centre	839
4	Chikuku	Rural Hospital	617
5	Bikita	Rural Hospital	902
6	Pfupajena	Rural Health Centre	540
7	Marozva	Rural Health Centre	389
8	Mukore	Rural Health Centre	285
9	Murwira	Rural Health Centre	284
10	Negovano	Rural Health Centre	296
11	Gangare	Rural Health Centre	261
12	Ngorima	Rural Health Centre	270
13	Mutikizizi	Rural Health Centre	216
14	Hozvi	Rural Health Centre	203
15	Chirorwe	Rural Health Centre	142
16	Chitasa	Rural Health Centre	146

Data are number and percentages of patients. Number of active ART patients according to SolidarMed ART (SMART) database by Dec 31, 2016.

6.2 Methods of minimising bias

6.2.1 Randomisation

Health facilities will be assigned in a 1:1 ratio to the intervention or the control group using a computer-generated, stratified blocked randomization. Randomization will be stratified by clinic size (<280, 280-1000, >1000 active ART patients). To avoid imbalance in the size of the groups, we will use blocked randomization within each stratum, with blocks consisting of two health facilities. The unit of random allocation will be the health facility and not the individual participant. This will minimize the risk of contamination between study arms. Randomization of health facilities will be done by a statistician who is not part of the study team at the Clinical Trials Unit in Bern, Switzerland. Randomization will be done prior to initiation of the recruitment phase.

6.2.2 Blinding procedures

Treatment assignment is unblinded to participants, lay health workers, health care providers, evaluators and data analysts.

6.2.3 Other methods of minimising bias

We will present the results from unadjusted analysis (accounting for clustering of data) and adjusted analysis. In adjusted analysis, we will control for the size of the clinic, age, gender, baseline self-reported adherence, type of ART regimen, and CD4 at baseline because randomization at the cluster-level may lead to imbalanced study arms.

7. STUDY POPULATION

7.1 Eligibility criteria

Participants eligible for the trial must comply with all of the following criteria at screening:

1. Age ≥18 years
2. On first-line antiretroviral therapy for at least 6 months
3. Resident in Bikita District
4. Speak and understand English or Shona
5. Able to comprehend the information on the study
6. Screened positive for CMDs (Shona Symptoms Questionnaire ≥9)
7. Provided written informed consent (consent by thumbprint) to participate study

Participants meeting any of the following criteria at screening will be excluded from the study:

1. Current psychosis / cognitive impairment
2. Clinical AIDS (WHO clinical stage 4)
3. Known pregnancy or ≤3 months postpartum

7.2 Recruitment and screening

At each study facility, research assistants will approach all persons in the waiting areas of ART clinics and ask them if they are willing to participate in a study on CMDs. Persons who agree to participate will be escorted to a suitable place for a confidential conversation (consultation room, bench in the outside area of the clinic). Research assistant will provide potential study participants with detailed information on the aim of the study and procedures. Research assistants will screen individuals, who have consented verbally, for CMDs and depression using the Shona Symptom Questionnaire (SSQ-14) and administer a brief screening questionnaire. Individual with a SSQ-14 score of 9 or higher who meet all other eligibility criteria will be included in the study after providing written consent. Participants who are not eligible for the study will be asked to provide verbal consent that their screening and questionnaire data may be used for statistical analysis to assess the prevalence of CMDs among rural HIV-infected population on antiretroviral therapy. Recruitment will continue until the target sample size is reached in each cluster.

7.3 Assignment to study groups

Assignment to study group will be done on clinic-level (cluster-level). For more details, see section 6.2.1.

7.4 Criteria for withdrawal / discontinuation of participants

Participants can withdraw their informed consent to participate in the study at any time. De-identified data that has been collected prior to participant withdrawal will be further used. Participant who withdraw consent will be referred to routine services and will be provided with a referral letter. Participants with severe mental illness who cannot be managed in the study facilities will be referred to psychiatric care. Analysis will be by intention to treat and participants will not be replaced. Sample size estimation accounts for dropouts.

8. STUDY INTERVENTION

8.1 Identity of Investigational Intervention

8.1.1 Experimental Intervention: Problem-Solving Therapy

Participants in the intervention group receive individual counselling and a group activity in addition to all services provided according to enhanced standard of care. Individual counselling is based on problem-solving therapy and delivered by lay health workers. Therapy sessions take place in a private area of the health facilities, which can be an outside area. Participants receive six counselling sessions of 30-45 minutes over a six-week period. Therapy takes a manualized structured approach to identify practical problems and teaches a positive attitude towards resolving them. Participants will be actively encouraged to identify and tackle problems leading to suboptimal adherence to ART. The structure of the intervention has been described in detail elsewhere.[22,24] After four session of individual counselling, participants are invited to join a peer-led group activity. In the group activity, participants are taught an income generating skill (e.g. to produce bags from recycled plastic) and have the opportunity to share personal experience with former participants of the interventoin.[22,24] Participants in the intervention group are also offered enhanced standard of care.

8.1.2 Control Intervention: Enhanced Standard of Care

In addition to the standard of care provided according to national ART guidelines, study participants receive information on available routine services for CMDs, a nurse-led brief support counseling, assessment for antidepressant medication (fluoxetine) by the clinic nurse, and referral to a psychiatric facility if needed. CD4 cell counts are measured at study entry. Viral load tests are done at recruitment, 24 weeks and 48 weeks. Adherence will be measure with electronic pill bottles (Medication Event Monitoring System [MEMS], AARDEX, Sion, Switzerland). Participants receive $3 transport reimbursement for each study visit and a reward of $10 for returning the electronic pill bottles at the end of the study.

8.1.3 Standard of Care for Patients on ART

According to national ART guidelines, all individuals with confirmed HIV infection are eligible for ART. Individuals receive a preferred fixed-dose first-line combination of once-daily tenofovir (TDF), lamivudine (3TC), and efavirenz (EFV) as preferred first-line regimen or one of the three alternative first-line regimens as per Zimbabwe National Guidelines. Alternative regimes are not available as one-pill fixed-dose combinations. Patients on ART are regularly seen at the facilities for clinic consultations and pharmacy refills. In general, they are seen every two weeks for the first month of treatment, thereafter monthly for three months, then every two to three months. After six months on ART, stable patient (viral load <1000 copies/ml or no current opportunistic infections and CD4 cell count ≥200 copies/ml) may be seen less frequently. The recommended monitoring strategy for patients on ART is viral load monitoring. Viral load should be measured routinely at 6 and 12 months after ART initiation, and then annually thereafter. CD4 cell count testing is measured at ART initiation and thereafter regularly in six-monthly intervals if viral load monitoring is not available. If VL monitoring is available, CD4 measurement is only done at baseline and on indication. Patients with viral load ≥1000 copies/ml receive enhanced adherence counselling and repeat viral load testing after 3 months of optimal ARV adherence. Patients who still have a viral load of ≥1000 copies/ml are switched to second-line ART. If viral load monitoring is not available, patients with suspected treatment failure should be switched based on immunological or clinical criteria. The second-line regimen is protease inhibitor-based and depending on first line regimen includes AZT/3TC or TDF/3TC.[28,29]

8.2 Compliance with Study Intervention

Research assistants will contact participants who missed clinic appointments or counselling sessions by phone call or home visit and encourage participants to return to care. Participants are asked for their informed consent to be contacted by home visit or phone call. Fidelity of lay health workers to problem-solving therapy manuals will be assessed and reinforced during weekly group supervision by a clinician or senior study team member trained in problem-solving therapy.

8.3 Data Collection and Follow-up for Withdrawn Participants

Participants who withdrew their consent to participate in the study will no longer be followed up. De-identified data that has been collected prior to participant withdrawal will be further used. The participants are offered to attend the last study visit. We will use multiple imputation procedures to

impute missing data from participants who withdrew informed consent. Participants referred to psychiatric care are asked to attend 3-monthly study visits to collect outcome data.

8.4 Concomitant Interventions (treatments)

All study participants undergo an assessment for antidepressant medication by the clinic nurse and will receive fluoxetine if indicated. Participants who cannot be managed within study facilities will be referral to a psychiatric facility and receive psychiatric care as indicated. Use of antidepressant medication and referral to psychiatric care will be documented in data collection tool.

9. STUDY ASSESSMENTS

9.1 Study flow charts of study procedures and assessments

Figure 3 shows a Gantt-chart of study procedures and assessments. At recruitment, we assess patients' eligibility for the study, measure HIV viral load, CD4 cell count, SSQ-14, and PHQ-9. At the first study visit (baseline), we measure will administer the baseline questionnaire. HIV viral load is again measured at 6 month and 12 month and SSQ-14, and PHQ-9 at 3, 6, 9 and 12 month. Adherence will be measured continuously from recruitment until the end of month 12. Participants in the intervention group receive six weekly lay health worker-led individual counselling session. Individual counselling begins at baseline or soon after (maximum time interval 3 days). After four sessions of individual counselling, participants in the intervention group are invited to join a group activity with six weekly per-led group support sessions. Participants in the interventions group receive one nurse-led individual counselling session at baseline or soon after. Study visits are within 3 days of recruitment and every three-month thereafter (Figure 1). Study visits will be aligned with participants' routine follow-up visits in the ART clinic.

Figure 3: Gantt-chart of showing participant timeline of counselling sessions and outcome assessments.

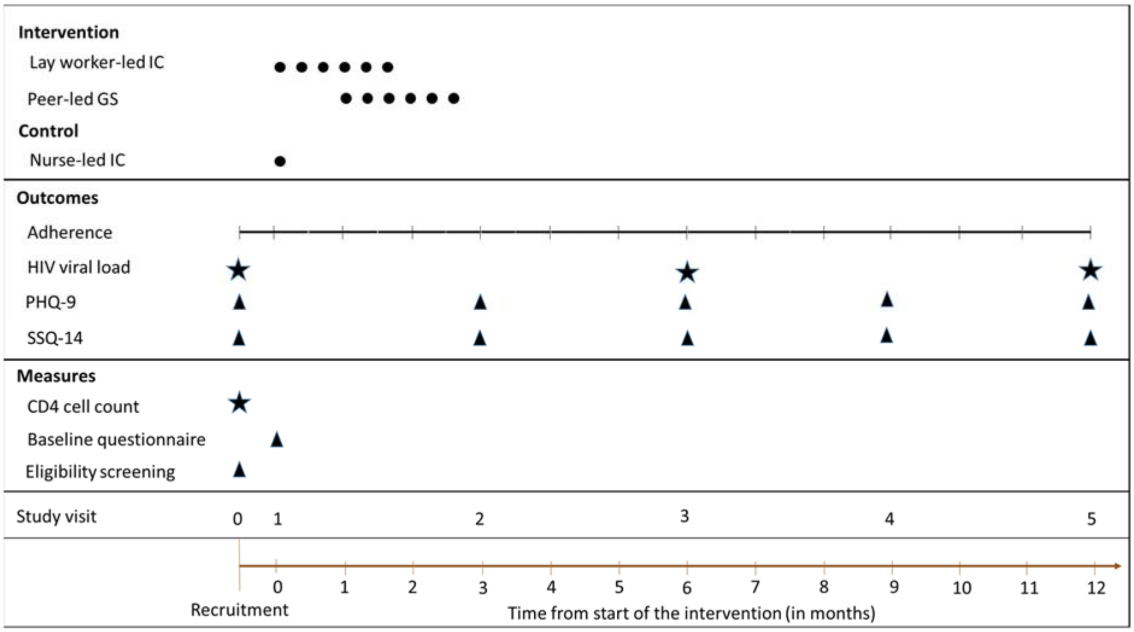

PST: Problem-solving therapy; MEMS: Medication Event Monitoring System; PHQ-9: Patient Health Questionnaire; SSQ-14: Shona Symptoms Questionnaire.

9.2 Assessments of outcomes

9.2.1 Assessment of primary outcome

We will use electronic pill bottles (Medication Event Monitoring System [MEMS], AARDEX, Sion, Switzerland) to measure the primary outcome average difference in mean adherence between 2-6 months. Study participants receive an electronic pill bottle at recruitment. They are advised to use pill bottles correctly and consistently during the entire study period. Research assistants extract adherence data from electronic pill bottles at each study visit and upload data to a cloud system. We will calculate participants' 4-weekly mean adherence scores as the percentage of doses taken per interval. For regimens that have to be taken once daily, a dose was considered as taken if the participant opens the electronic pill bottle within -8 to +16 hours of the designated dosing time. For regimens that have to be taken twice daily, a dose was considered as taken if the participant opens the electronic pill bottle within -4 to +8 hours of the designated dosing time for patients. There is no gold standard adherence measure. We chose a MEMS-based adherence measures as primary outcome because this measure has been shown to be more accurate than self-reported adherence.[30] We could not use a pharmacy-based adherence measure because detailed pharmacy data during routine care.[31]

9.2.2 Assessment of secondary outcomes

We will use the same methods as for the primary outcomes to measure the secondary outcome average difference in mean adherence between 1-12 months. We will measure the secondary outcomes change from baseline in SSQ-14 and PHQ-9 score at 6 month and 12 month using English or Shona versions of the screening tools. Both screening tools have been validated in Zimbabwe in English and Shona language and showed a high sensitivity and specificity against diagnosis of depression and general anxiety disorder.[23] We will use viral load testing to assess the secondary outcome change from baseline in proportion of participants with a viral load suppression (<20 copies per milliliter) at 6 months and 12 months. VL test samples will be collected from the study participants via venepuncture by the nurses at the ART clinic in ethylenediaminetetraacetic acid tubes and send to Silveira district laboratory using the existing Ministry of Health specimen transport system. Within 5 days, the cell free ethylenediaminetetraacetic acid plasma samples will be transported to Masvingo provincial laboratory where the samples will be tested on the existing Roche platform. Results will be sent back to the district laboratory on line using a secured link.

9.2.3 Assessment of eligibility criteria, baseline and other outcome measures

Research assistants will extract data from medical records and administer a brief screening questionnaire and the SSQ-14 to assess eligibility of study participants. Research assistants will administer questionnaires and screening tools to collect baseline and outcome measurements. We will use items from the Demographic and Health Survey (DHS) and AIDS indicator survey (AIS) to assess socio-demographic characteristics.[32] We will use items form the DHS, AIS, the Zimbabwe Population-Based HIV Impact Assessment (ZIMPHIA), and the Patient–Provider Relationship Scale (PPRS) to measure HIV-related variables, gender norms, and health-system indicators.[32–35] Alcohol misuse will be assessed with the AUDID-C, food insecurity with a subset of the Household Food Insecurity scale (HFIS) and social support with the eight-item Medical Outcomes Study Social Support Survey (MOS-SS).[36–38] Participants' general health perception is measured with selected items from the Medical Outcomes Study Short Form Survey Instrument (MOS SF-36).[39] We will use CD4 testing to assess participants CD4 at recruitment. CD4 samples will be processed at the district laboratory within 24 hours and results communicated back to the ART clinics using the Ministry of Health specimen transport system.

9.2.4 Assessment of safety outcomes

Patients who were referred to psychiatric care will be asked to attend 3-monthly follow-up visits. Research assistants will administer a questionnaire to assess whether participants were admitted to a psychiatric hospital or were had a suicide attempt. Participants who do not attend 3-monthly follow-up visits will be contacted by home visit or phone calls and study instruments will be administered during home visits if patients do not wish to attend study visits. If deemed necessary, the local-PI or designated project staff will conduct a verbal autopsy to assess whether a participant committed suicide using the 2016 WHO verbal autopsy instrument.[40]

9.2.5 Assessments in participants who prematurely stop the study

Participants referred to psychiatric care are asked to attend 3-monthly study visits to collect data on all primary and secondary outcomes. Participants who withdrew their consent to participate in the study are offered to attend the last study visit and keep the electronic pill bottle. All other study assessments will be omitted.

Study visits are within 3 days of recruitment and every three-month thereafter. Study visits will be aligned with participants' routine follow-up visits in the ART clinic if possible.

9.2.6 Recruitment

At recruitment (visit 0), we assess patients' eligibility for the study, measure HIV viral load, CD4 cell count, SSQ-14, and PHQ-9 (Figure 3).

9.2.7 Baseline assessment (visit 1)

At the first study visit (baseline), we measure will administer the baseline questionnaire (Figure 3).

9.2.8 Follow-up visits

At the 3-monthly follow-up visits (visits 2-5), we measure HIV viral load and mental health outcomes. HIV viral load is again measured at 6 month and 12 month and SSQ-14, and PHQ-9 at 3, 6, 9 and 12 month (Figure 3).

9.2.9 Adherence measurement

Adherence will be measured continuously from recruitment until the end of month 12. Research assistants will extract data from electronic pill bottles at study visits 1-5 and upload data into the cloud system (Figure 3). Advice on correct and consistent usage of electronic pill bottles is repeated at each study visit.

9.2.10 Intervention group: problem-solving therapy and group support visits

Participants in the intervention group receive six weekly lay health worker-led individual counselling session. Individual counselling begins at baseline or soon after (maximum time interval 3 days). After four sessions of individual counselling, participants in the intervention group are invited to join a group activity with six weekly per-led group support sessions (Figure 3). Lay health workers will document attendance of participants at individual counselling session and covered content in dedicated logbooks. Facilitators or peer support groups document attendance at group support sessions using attendance lists. A random sample of individual counselling sessions will be tape recorded for qualitative assessment of fidelity to counselling manuals.

9.2.11 Control group: enhanced standard of care visits

Participants in the interventions group receive one nurse-led individual counselling session at baseline or soon after (Figure 3).

10. SAFETY

During the entire duration of the study and all serious adverse events (SAEs) are collected, fully investigated and documented in source documents and case report forms (CRF). Study duration encompassed the time from when the participant signs the informed consent until the last protocol-specific procedure has been completed, including a safety follow-up period.

10.1 Definition and assessment of serious adverse events

A **Serious Adverse Event** is classified as any untoward medical occurrence that:

- results in death,
- is life-threatening,
- requires in-patient hospitalization or prolongation of existing hospitalisation,
- results in persistent or significant disability/incapacity

In addition, important medical events that may not be immediately life-threatening or result in death, or require hospitalisation, but may jeopardise the patient or may require intervention to prevent one of the other outcomes listed above should also usually be considered serious.

The following events will be considered SAEs: psychiatric hospitalization, suicide attempt, or suicide. This list is non-exhaustive.

SAEs should be followed until resolution or stabilisation. Participants with ongoing SAEs at study termination (including safety visit) will be further followed up until recovery or until stabilisation of the disease after termination.

Assessment of Causality

Both Investigator and Sponsor-investigator make a causality assessment of the event to the psychological intervention, based on the criteria listed in the ICH E2A guidelines:

Relationship	Description
Definitely	Temporal relationship<br>Improvement after dechallenge*<br>Recurrence after rechallenge<br>(or other proof of drug cause)
Probably	Temporal relationship<br>Improvement after dechallenge<br>No other cause evident
Possibly	Temporal relationship<br>Other cause possible
Unlikely	Any assessable reaction that does not fulfil the above conditions
Not related	Causal relationship can be ruled out
*Improvement after dechallenge only taken into consideration, if applicable to reaction	

10.2 Reporting of serious adverse events

Serious adverse events will be reported to local authorities in Zimbabwe within three days of awareness using the standard reporting form provided by Medicines Control Authority of Zimbabwe, as is the practice in all Ministry of Health and Child Care institutions.

11. STATISTICAL METHODS

11.1 Hypothesis

We will test the null hypothesis that there is no difference in mean adherence during months 2-6 between study arms. Our alternative hypothesis is that there is a difference in mean adherence during months 2-6 between study arms.

11.2 Determination of Sample Size

Sample size and power calculation for two independent means in a cluster-randomized design was done in PASS (Version 14, NCSS, LLC. Kaysville, Utah, USA). To detect differences in means a two-sided t-test was used with a significance level of 0.050. This test used degrees of freedom based on the number of subjects.[41–43]

Assuming an intra-cluster coefficient (ICC) of 0.05, a sample size of 400 participants (16 clusters with 25 participants per cluster) provides 92% power to detect a moderate effect size (mean difference: 10%; standard deviation [SD]: 20; standardized mean difference [SMD]: 0.5), (Figure 5). The assumption of the effect size was based on a recent randomized trial on the effect of problem-solving therapy on adherence.[19] The ICC was conservatively estimated based on a study from Tanzania which showed ICC of most HIV outcomes were below 0.02.[44]. To account for loss to follow-up and incomplete data, we will recruit 480 participants (16 clusters with each 30 participants), assuming retention in care and complete data for at least 83% of participants (25/30 participants per cluster).

Figure 5: Statistical power to detect differences in two independent means.

Graph shows the statistical power (pwr) to detect a medium effect size (Cohen's d of 0.5) for a sample size of each 6, 7, 8, 9, 10, 11, and 12 clusters of 25 participants (M) in the experimental (K1) and control condition (K2), assuming a type I error of 5%, and an intra-cluster coefficient (ICC) of 0.05.

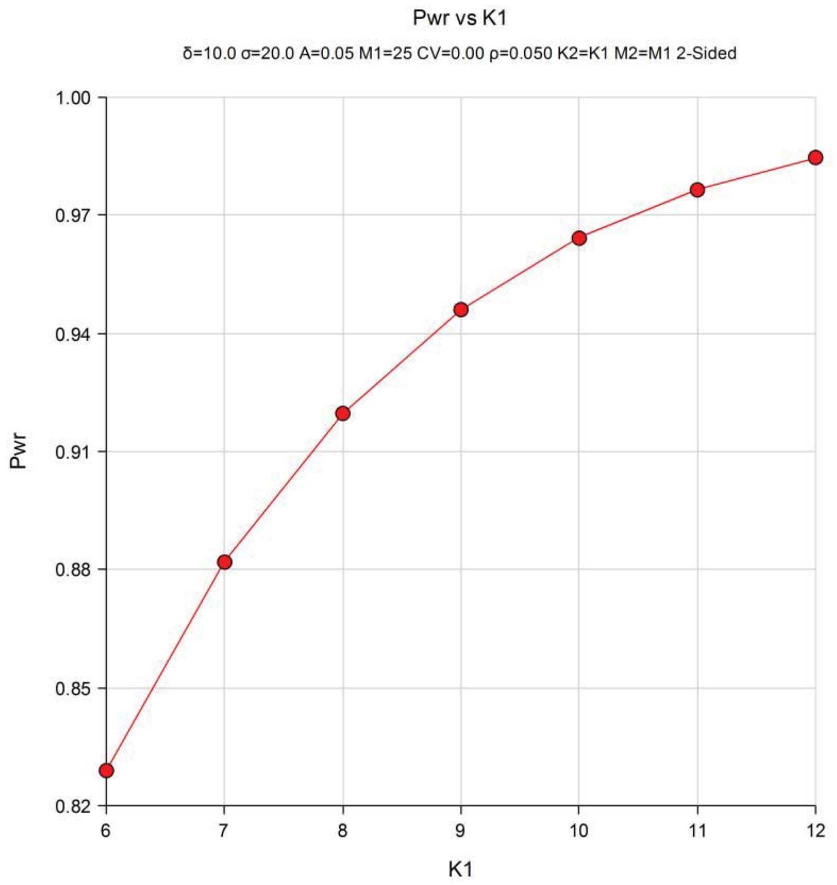

In a sensitivity analysis, we examined statistical power for different effect sizes and different ICCs for a sample size of 16 clusters with 25 participants in each cluster. Assuming an ICC of 0.02, a moderate to weak effect size (mean difference: 7%; SD: 20; SMD: 0.35) provides 82% power to detect differences in two independent means. Assuming an ICC of 0.1, a moderate effect size (mean difference: 11%; SD: 20; SMD: 0.55) provides 84% statistical power to detect differences in two independent means (Figure 6).

Figure 6: Statistical power to detect differences in two independent means for different effect sizes and different intra-cluster coefficients.

Graph shows the statistical power (pwr) to detect two differences in in two independent means in a sample of each 8 clusters (K) of 25 participants (M) in the control and experimental arm for different mean differences (δ) and difference intra-cluster coefficient (ρ).

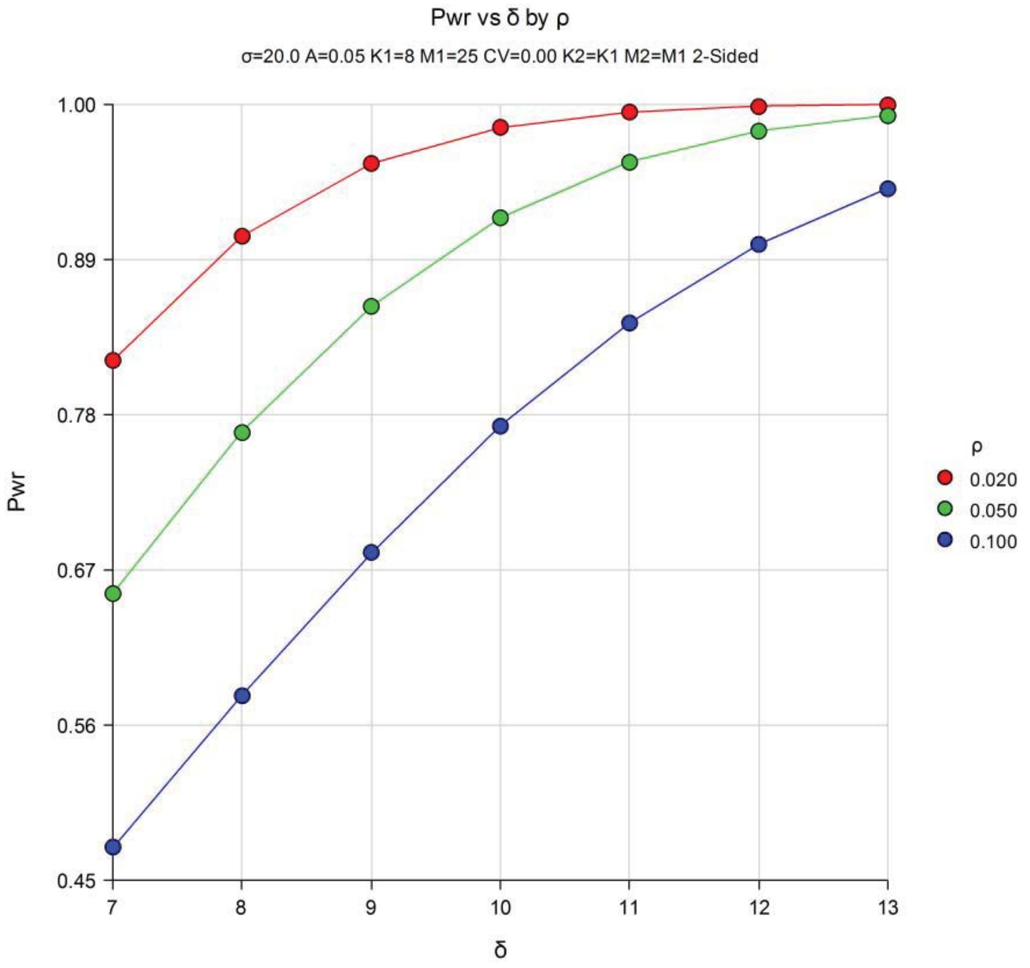

11.3 Planned Analyses

The intervention group (problem-solving therapy) will be compared against the control group (enhanced standard of care) in all analyses. The intervention effect will be assessed based by intention-to-treat analysis. The intervention effect will be assessed based by intention-to-treat analysis. Analysis time will be measured in months since the first scheduled study visits. We will use descriptive analysis, and logistic and linear mixed effects models. All statistical analyses will be done in Stata 14.0

11.3.1 Datasets to be analysed, analysis populations

We will analyze three different datasets:

1. Dataset with data on individuals who were assessed for study eligibility. The dataset includes variables collected with eligibility screening tool and the SSQ-14 scores.
2. Dataset with data on individuals who participated in the study. The dataset includes variables collected with the baseline questionnaire. adherence data, laboratory results (CD4 and viral load test) and mental health assessments (SSQ-14, and PHQ-9)
3. Dataset with data on serious adverse events and other safety relevant outcomes.

11.3.2 Primary Analysis

We will use linear mixed effect models to assess the average difference in mean adherence. Models include indicators for random intercept and slope on participant-level to account for correlation of measurements within participants, an indicator for random intercept on cluster-level to account for clustering of individuals in health facilities, an indicator for treatment assignment, an indicator for each months of analysis time, and interactions of treatment assignment and each months of analysis time. We will present the results from unadjusted analysis (accounting for clustering of data) and adjusted analysis. In adjusted analysis, we will control for the size of the clinic, age, gender, baseline self-reported adherence, type of ART regimen, and CD4 at baseline.

We will use linear mixed effect models to assess the difference in change from baseline in SSQ-14 and PHQ-9 score. Models include indicators for random intercept and slope, an indicator for random intercept on cluster-level, an indicator for treatment assignment, an indicator for analysis time, and an interaction of treatment assignment and analysis time. We will present results from unadjusted analysis (accounting for clustering of data) and adjusted analysis. In adjusted analysis, we will control for the size of the clinic, age, and gender.

We will use logistic mixed effect models to assess the difference in the proportion of participants with viral load suppression at 6 month. Models an indicator for random intercept on cluster-level, and an indicator for treatment assignment. We will present results from unadjusted analysis (accounting for clustering of data) and adjusted analysis. In adjusted analysis, we will control for the size of the clinic, age, and gender and baseline viral load suppression.

We will use linear mixed effect models to assess the difference in change from baseline in the proportion of participants with viral load suppression at 12 month. Models include indicators for random intercept and slope, an indicator for random intercept on cluster-level, an indicator for treatment assignment, an indicator for analysis time, and an interaction of treatment assignment and analysis time. We will present results from unadjusted analysis (accounting for clustering of data) and adjusted analysis. In adjusted analysis, we will control for the size of the clinic, age, and gender.

Analyses will be done after follow-up has been completed in all study facilities. Analyses will be done at the Institute of Social and Preventive Medicine and Clinical Trials Unit at University of Bern.

11.3.3 Secondary Analyses

Secondary analysis will be described in detail in a statistical analysis plan.

11.3.4 Safety analysis

We will calculate unadjusted incidence rates stratified by study arm and unadjusted incidence rate ratios to describe safety outcomes.

11.3.5 Deviation(s) from the original statistical plan

Deviations from the final statistical analysis plan will be justified and reported in publications.

11.4 Handling of missing data and drop-outs

We will impute missing data using multiple imputation with chained equations.[45]

12. QUALITY ASSURANCE AND CONTROL

The sponsor and principal investigators will develop written SOPs for all study procedures and train study personal. Dr. Chibanda, from the Department of Psychiatry, University of Zimbabwe, overseas training and supervision of lay health workers and implementation of the friendship bench intervention.

12.1 Data handling and record keeping / archiving

The Investigators will maintain appropriate research records for this trial, in compliance with ICH-GCP and regulatory and institutional requirements for the protection of confidentiality of subjects. The Principal Investigator, Sub-investigator, and Clinical Research Nurses or Coordinators will have access to the records. The Principal Investigators will permit authorized representatives of the Sponsor and regulatory agencies to examine clinical records for the purposes of quality assurance reviews, audits, and evaluation of the study safety and progress.

12.1.1 Case Report Forms

A unique study identifier will be used to identify patients on all data collection forms. Research assistants will use tablet computers with custom-built data collection forms to collect study data. The Investigator should ensure the accuracy, and completeness of the data reported in the eCRF and all other required reports. Only designated study personal can login data collection systems using a personal username and password.

12.1.2 Specification of source documents

A record with participants study identifier and participants ART registration number is considered being source data. Source data remains with the research assistant at the respective study facility. Routinely collected data on antiretroviral therapy of the participant remains with the antiretroviral therapy provider and can be linked to the participant using the ART registration number.

12.1.3 Record keeping / archiving

All study data (written and electronic), will be archived by the local principal investigator at the SolidarMed Zimbabwe office in Zimbabwe for at least 10 year. The Investigators should take measures to prevent accidental or premature destruction of these documents.

12.2 Data management

A unique study identifier will be used to identify patients on all data collection forms. Research assistants will keep a list of that is linking unique study identifiers and personal information of the person enrolled at the study facility to be able to find out the unique study identifiers of participants who forgot their study identifier number. Research assistants will keep this list strictly confidential. Research assistants use tablet computers with custom-built data collection forms to abstract data from medical records and collect questionnaire data. Data collection tools apply real-time data validation rules. Data from tablets will be uploaded to an Open Data Kit (ODK) server at weekly intervals, ODK is a secure and free and open-source tools for mobile data collection. Data will be exported from ODK server at weekly intervals for error checking, and reporting. Data will be backed-up on an external hard-drive weekly. Research assistants will use tablet computers to read adherence data from electronic pill bottles. Data from tablets will be uploaded to medAmigo, a secure cloud-based data platform that is provided by the manufacturer of the electronic pill bottles. Data will be exported from the medAmigo server at two-weekly intervals for error checking, and reporting. All data will be kept strictly confidential, under lock-and-key. Data are only accessible by designated study staff on password-protected computers.

12.2.1 Data Management System

The electronic data capture (EDC) system is activated for the trial only after successfully passing a formal test procedure. Research assistants use tablet computers with custom-built data collection forms to abstract data from medical records and collect questionnaire data. Data collection tools apply real-time data validation rules. Data from tablets will be uploaded to an Open Data Kit (ODK) server at weekly intervals, ODK is a secure and free and open-source tools for mobile data collection. Research assistants will use tablet computers to read adherence data from electronic pill bottles. Data from tablets will be uploaded to medAmigo, a secure cloud-based data platform that is provided by the manufacturer of the electronic pill bottles. Responsibility for hosting the EDC system and the database lies with SolidarMed.

12.2.2 Data security, access and back-up

Data will be exported from ODK server at weekly intervals for error checking, and reporting. The server hosting the EDC system and the database is kept in a locked server-room. Only the system administrators have direct access to the server. Data will be backed-up on an external hard-drive weekly. The hard-drives will be kept locked. Research assistants will use tablet computers to read adherence data from electronic pill bottles. Data from tablets will be uploaded to medAmigo, a secure cloud-based data platform that is provided by the manufacturer of the electronic pill bottles. Data will be exported from the medAmigo server at two-weekly intervals for error checking, and reporting. All data will be kept strictly confidential, under lock-and-key. Data are only accessible by designated study staff on password-protected computers.

12.2.3 Analysis and archiving

During the study, data will be regularly exported and backed up from cloud systems and backups will be stored at the SolidarMed Zimbabwe office and the Institute of Social and Preventive Medicine. Final analyses, data files will be extracted from the database into statistical packages to be analyzed. The status of the database at this time is recorded in special archive tables.
Data will be archived at the SolidarMed Zimbabwe office and at the Institute of Social and Preventive Medicine, University of Bern for at least 10 years. Corresponding authors of publications keep a copy of any data that was used in publications for at least 10 years.

12.2.4 Electronic and central data validation

Data collection tools apply real-time data validation rules. Data will be exported from cloud systems in weekly intervals for error checking, and reporting.

12.3 Monitoring

A delegated and trained person (central data monitor) will export data from cloud systems in regular intervals for central data monitoring purposes. The central data monitor will continuously monitor completeness of data collection and data quality (i.e. systematic data validation and plausibility checks). Central data monitors will send summaries with incomplete or implausible data to the field team for further investigation and correction (if needed).

A delegated and trained person (monitor) will visit randomly chosen study sites to control the study conduct and data retrieval. Any findings and comments will be documented in site visit reports and communicated to the local Investigator and to the Sponsor as applicable. Investigators at the participating study sites will support the Monitor in his/ her activities. Prior to study start (first participant enrolled) a plan detailing all monitoring-related procedures will be developed. All source data and relevant documents will be accessible to Monitors and questions of Monitors are answered during site visits. Any monitoring activities will be described in the data management and validation plan.

12.4 Audits and Inspections

Principal investigators will conduct unannounced random audits and review adherence to SOPs, data collection, and storage of blood samples. All study and source documents are accessible to auditors. The CA (Swissmedic) or CEC may wish to conduct an inspection (during the study or after its completion). If an inspection is requested, the Investigator must inform the Sponsor immediately that this request has been made. The Investigators at the participating sites will support the inspectors in their activities and will answer questions from inspectors as needed. All involved parties must keep the participant data strictly confidential.

12.5 Confidentiality, Data Protection

All data will be kept strictly confidential, under lock-and-key. Data are only accessible by designated study staff on password-protected computers. The research coordinator will assign a unique study identifier to each participant. The list that is linking unique study identifiers and personal information such as names, and date of birth will be kept strictly confidential by the research coordinator on encrypted drives and separately from other project data.

12.6 Storage of biological material and related health data

Within 5 days, the cell free ethylenediaminetetraacetic acid plasma samples will be transported to Masvingo provincial laboratory where the samples will be tested using routine laboratory services. Samples will be destroyed subsequently.

13. PUBLICATION AND DISSEMINATION POLICY

The study will provide data on the implementation of the Friendship Bench intervention at rural health facilities in Bikita District, for the management of common mental health disorders among HIV patients. Based on the findings, recommendations can be made on how to optimize mental health service delivery at primary health care level in comparable settings within Zimbabwe. Findings will be communicated directly to health care providers and health policy specialists. Study results will be presented at stakeholders meetings, including Ministry of Health and Child Care, and will be shared in presentations and publications, both within Zimbabwe and internationally. We will make an effort to share the data in a timely fashion with the MoHCC. We will aim to present study results at international conferences (CROI, IAS, AIDS) and publish journal articles in international peer-reviewed journals.

14. FUNDING AND SUPPORT

14.1 Funding

The study is funded by the National Institute of Allergy and Infectious Diseases, the National Institute of Child Health and Human Development and the National Cancer Institute and the National Institute of Mental Health of the US National Institutes of Health through a grant to the International epidemiology Databases to Evaluate AIDS Southern Africa under the award number U01AI069924 (PIs: Egger & Davies).

15. INSURANCE

Insurance will be provided by the Sponsor. A copy of the certificate is filed in each investigator site file and the trial master file.

16. REFERENCES

1 Joint United Nations Programme on HIV/AIDS (UNAIDS). AIDSInfo online database. http://www.aidsinfoonline.org/devinfo/libraries/aspx/dataview.aspx (accessed Dec 27, 2017).

2 Chibanda D, Cowan F, Gibson L, Weiss HA, Lund C. Prevalence and correlates of probable common mental disorders in a population with high prevalence of HIV in Zimbabwe. *BMC Psychiatry* 2016; **16**: 55.

3 Ministry of Health and Child Care Z. Guidelines for Antiretroviral Therapy for the Prevention and Treatment of HIV in Zimbabwe. 2013. http://www.nac.org.zw/sites/default/files/2013 Zimbabwe ARV Guidelines Main Document (1).pdf (accessed March 2, 2017).

4 Nachega JB, Hislop M, Dowdy DW, Chaisson RE, Regensberg L, Maartens G. Adherence to Nonnucleoside Reverse Transcriptase Inhibitor – Based HIV Therapy and Virologic Outcomes. *Ann Intern Med* 2007; : 564–73.

5 Chi BH, Cantrell R a, Zulu I, Mulenga LB, Levy JW, Tambatamba BC, Reid S, Mwango A, Mwinga A, Bulterys M, Saag MS, Stringer JS a. Adherence to first-line antiretroviral therapy affects non-virologic outcomes among patients on treatment for more than 12 months in Lusaka, Zambia. *Int J Epidemiol* 2009; **38**: 746–56.

6 Nachega JB, Hislop M, Dowdy DW, Lo M, Omer SB, Regensberg L, Chaisson RE, Maartens G. Adherence to highly active antiretroviral therapy assessed by pharmacy claims predicts survival in HIV-infected South African adults. *J Acquir Immune Defic Syndr* 2006; **43**: 78–84.

7 Sethi AK, Celentano DD, Gange SJ, Moore RD, Gallant JE. Association between adherence to antiretroviral therapy and human immunodeficiency virus drug resistance. *Clin Infect Dis* 2003; **37**: 1112–8.

8 Mannheimer S, Friedland G, Matts J, Child C, Chesney M. The consistency of adherence to antiretroviral therapy predicts biologic outcomes for human immunodeficiency virus-infected persons in clinical trials. *Clin Infect Dis* 2002; **34**: 1115–21.

9 Cohen MS, Chen YQ, McCauley M, Gamble T, Hosseinipour MC, Kumarasamy N, Hakim JG, Kumwenda J, Grinsztejn B, Pilotto JHS, Godbole S V, Mehendale S, Chariyalertsak S, Santos BR, Mayer KH, Hoffman IF, Eshleman SH, Piwowar-Manning E, Wang L, *et al.* Prevention of HIV-1 infection with early antiretroviral therapy. *N Engl J Med* 2011; **365**: 493–505.

10 Nakimuli-Mpungu E, Bass JK, Alexandre P, Mills EJ, Musisi S, Ram M, Katabira E, Nachega JB. Depression, alcohol use and adherence to antiretroviral therapy in sub-Saharan Africa: A systematic review. *AIDS Behav* 2012; **16**: 2101–8.

11 Uthman OA, Magidson JF, Safren SA, Nachega JB. Depression and adherence to antiretroviral therapy in low-, middle- and high-income countries: a systematic review and meta-analysis. *Curr HIV/AIDS Rep* 2014; **11**: 291–307.

12 Krumme AA, Kaigamba F, Binagwaho A, Murray MB, Rich ML, Franke MF. Depression, adherence and attrition from care in HIV-infected adults receiving antiretroviral therapy. *J Epidemiol Community Health* 2015; **69**: 284–9.

13 Pence BW, Miller WC, Gaynes BN, Eron JJ. Psychiatric illness and virologic response in patients initiating highly active antiretroviral therapy. *J Acquir Immune Defic Syndr* 2007; **44**: 159–66.

14 Adewuya AO, Afolabi MO, Ola BA, Ogundele OA, Ajibare AO, Oladipo BF. Psychiatric disorders among the HIV-positive population in Nigeria: A control study. *J Psychosom Res* 2007; **63**: 203–6.

15 Pyne JM, Fortney JC, Curran GM, Tripathi S, Atkinson JH, Kilbourne AM, Hagedorn HJ, Rimland D, Rodriguez-Barradas MC, Monson T, Bottonari KA, Asch SM, Gifford AL. Effectiveness of Collaborative Care for Depression in Human Immunodeficiency Virus Clinics. *Arch Intern Med* 2010; **171**: 23–31.

16 Pence BW, Gaynes BN, Adams JL, Thielman NM, Heine AD, Mugavero MJ, McGuinness T, Raper JL, Willig JH, Shirey KG, Ogle M, Turner EL, Quinlivan EB. The effect of antidepressant treatment on HIV and depression outcomes: results from a randomized trial. *AIDS* 2015; **29**: 1975–86.

17 Tsai AC, Karasic DH, Hammer GP, Charlebois ED, Ragland K, Moss AR, Sorensen JL, Dilley JW, Bangsberg DR. Directly observed antidepressant medication treatment and HIV outcomes among homeless and marginally housed HIV-positive adults: A randomized controlled trial. *Am J Public Health* 2013; **103**: 308–15.

18 Safren SA, O'Cleirigh CM, Bullis JR, Otto MW, Stein MD, Pollack MH. Cognitive behavioral therapy for adherence and depression (CBT-AD) in HIV-infected injection drug users: A randomized controlled trial. *J Consult Clin Psychol* 2012; **80**: 404–15.

19 Safren SA, Bedoya CA, O'Cleirigh C, Biello KB, Pinkston MM, Stein MD, Traeger L, Kojic E, Robbins GK, Lerner JA, Herman DS, Mimiaga MJ, Mayer KH. Cognitive behavioural therapy for adherence and depression in patients with HIV: a three-arm randomised controlled trial. *Lancet HIV* 2016; **3**: e529–38.

20 Safren S a, O'Cleirigh C, Tan JY, Raminani SR, Reilly LC, Otto MW, Mayer KH. A randomized controlled trial of cognitive behavioral therapy for adherence and depression (CBT-AD) in HIV-infected individuals. *Health Psychol* 2009; **28**: 1–10.

21 Simoni JM, Wiebe JS, Sauceda JA, Huh D, Sanchez G, Longoria V, Andres Bedoya C, Safren SA. A preliminary RCT of CBT-AD for adherence and depression among HIV-positive latinos on the U.S.-Mexico border: The Nuevo Día study. *AIDS Behav* 2013; **17**: 2816–29.

22 Chibanda D, Weiss HA, Verhey R, Simms V, Munjoma R, Rusakaniko S, Chingono A, Munetsi E, Bere T, Manda E, Abas M, Araya R. Effect of a Primary Care–Based Psychological Intervention on Symptoms of Common Mental Disorders in Zimbabwe. *JAMA* 2016; **316**: 2618.

23 Chibanda D, Verhey R, Gibson LJ, Munetsi E, Machando D, Rusakaniko S, Munjoma R, Araya R, Weiss HA, Abas M. Validation of screening tools for depression and anxiety disorders in a primary care population with high HIV prevalence in Zimbabwe. *J Affect Disord* 2016; **198**: 50–5.

24 Chibanda D, Bowers T, Verhey R, Rusakaniko S, Abas M, Weiss HA, Araya R. The Friendship Bench programme: A cluster randomised controlled trial of a brief psychological intervention for common mental disorders delivered by lay health workers in Zimbabwe. *Int J Ment Health Syst* 2015; **9**: 1–7.

25 Patel V, Simunyu E, Gwanzura F. Kufungisisa (thinking too much): a Shona idiom for non-psychotic mental illness. *Cent Afr J Med* 1995; **41**: 209–15.

26 Solidarmed Zimbabwe. http://www.solidarmed.ch/de/laender/zimbabwe (accessed March 3, 2017).

27 Egger M, Ekouevi DK, Williams C, Lyamuya RE, Mukumbi H, Braitstein P, Hartwell T, Graber C, Chi BH, Boulle A, Dabis F, Wools-Kaloustian K. Cohort Profile: the international epidemiological databases to evaluate AIDS (IeDEA) in sub-Saharan Africa. *Int J Epidemiol* 2012; **41**: 1256–64.

28 Ministry of Health and Child Care Z. Guidelines for Antiretroviral Therapy for the Prevention and Treatment of HIV in Zimbabwe. 2013.

29 Guidelines for Antiretroviral Therapy for the Prevention and Treatment of HIV in Zimbabwe. https://aidsfree.usaid.gov/sites/default/files/zw_arv_therapy_prevention_updated.pdf.

30 Vitolins MZ, Rand CS, Rapp SR, Ribisl PM, Sevick MA. Measuring Adherence to Behavioral and Medical Interventions. *Control Clin Trials* 2000; **21**: S188–94.

31 McMahon JH, Jordan MR, Kelley K, Bertagnolio S, Hong SY, Wanke C a., Lewin SR, Elliott JH. Pharmacy Adherence Measures to Assess Adherence to Antiretroviral Therapy: Review of the Literature and Implications for Treatment Monitoring. *Clin Infect Dis* 2011; **52**: 493–506.

32 DHS. Demographic and Health Survey (DHS). 2017. https://dhsprogram.com/What-We-Do/Survey-Types/DHS.cfm.

33 AIS. AIDS Indicator Survey (AIS). 2017. https://dhsprogram.com/What-We-Do/Survey-Types/AIS.cfm.

34 ZIMPHIA. Zimbabwe Population-based HIV Impact Assessment. 2016. http://phia.icap.columbia.edu/wp-content/uploads/2016/11/ZIMBABWE-Factsheet.FIN_.pdf (accessed Aug 4, 2017).

35 Barry OM, Bergh A-M, Makin JD, Etsane E, Kershaw TS, Forsyth BWC. Development of a measure of the patient-provider relationship in antenatal care and its importance in PMTCT. *AIDS Care* 2012; **24**: 680–6.

36 Bradley KA, Debenedetti AF, Volk RJ, Williams EC, Frank D, Kivlahan DR. AUDIT-C as a brief screen for alcohol misuse in primary care. *Alcohol Clin Exp Res* 2007; **31**: 1208–17.

37 Coates J, Swindale A, P B. Household Food Insecurity Access Scale (HFIAS) for Measurement of Household Food Access: Indicator Guide (v.3). Washington, DC: : Food and Nutrition Technical Assistance Project, 2007

http://journals.cambridge.org/download.php?file=%2FPHN%2FPHN18_15%2FS1368980015000403a.pdf&code=f90b2966d763fb54968140bc4db80943.

38 Moser A, Stuck AE, Silliman RA, Ganz PA, Clough-Gorr KM. The eight-item modified Medical Outcomes Study Social Support Survey: Psychometric evaluation showed excellent performance. *J Clin Epidemiol* 2012; **65**: 1107–16.

39 Medical Outcomes Study. SF-36. https://www.rand.org/health/surveys_tools/mos/36-item-short-form.html.

40 World Health Organization. 2016 WHO verbal autopsy instrument. http://www.who.int/healthinfo/statistics/verbalautopsystandards/en/ (accessed Jan 23, 2018).

41 Campbell M., Walters S. How to Design, Analyse and Report Cluster Randomised Trials in Medicine and Health Related Research. New York: Wiley, 2014.

42 Ahn C, Heo M, Zhang S. Sample Size Calculations for Clustered and Longitudinal Outcomes in Clinical Research. New York: CRC Press, 2015.

43 Donner A, Klar N. Design and Analysis of Cluster Randomization Trials in Health Research. London: Arnold, 2000.

44 Barnhart D, Hertzmark E, Liu E, Mungure E, Muya AN, Sando D, Chalamilla G, Ulenga N, B??rnighausen T, Fawzi W, Spiegelman D. Intra-cluster correlation estimates for HIV-related outcomes from care and treatment clinics in Dar es Salaam, Tanzania. *Contemp Clin Trials Commun* 2016; **4**: 161–9.

45 Carpenter J, Kenward M. Multiple Imputation and its Applicatoin. West Sussex, UK: John Wiley & Sons, Ltd, 2013.

Statistical Analysis Plan (SAP)

Effect of a psychological intervention on antiretroviral therapy outcomes and symptoms of common mental disorders in HIV-positive adults in rural Zimbabwe: cluster-randomized trial

Friendship Bench Trial

Administrative Information

Project number:	794
Trial registration number:	NCT03704805
SAP version:	Version 1.0, April 29, 2019
Protocol version:	Version 4, April 05, 2018

Contributors

Name	Affiliation	Role in SAP writing
Andreas Limacher	CTU Bern	Author
Stefanie Hossmann	CTU Bern	Author
Andreas Haas	ISPM Bern	Reviewer

Revision history

Revision	Justification	Timing
n.a. (first version)		

Approved by

Name	Affiliation	Study Role	Date and Signature (wet ink)
Andreas Haas	ISPM Bern	Principal Investigator, Trial Statistician	06/05/19
Andreas Limacher	CTU Bern	Senior Statistician	6.5.19
Stefanie Hossmann	CTU Bern	Clinical Trial Manager	6.5.19

Contents

1. Introduction 6

1.1 Background and rationale/ 6

1.2 Objectives 6

2. Study methods 7

2.1 Trial design 7

2.2 Randomization 7

2.3 Sample size 7

2.4 Framework 7

2.5 Statistical interim analyses and stopping guidance 7

2.6 Timing of final analysis 7

2.7 Timing of outcome assessments 7

2.8 Blinding 7

3. Data management 8

3.1 Data export 8

3.2 Data validation 8

3.3 Data preparation 8

3.3.1 Adherence data (MEMS data) 8

3.3.2 SSQ-14 8

3.3.3 PHQ-9 8

3.3.4 Viral load suppression 8

3.3.5 Safety outcomes 9

3.3.6 Data sharing 9

4. Statistical principles 10

4.1 Confidence intervals and *P* values 10

4.2 Analysis populations 10

4.2.1 Full analysis set (FAS) 10
4.2.2 Per-protocol (PP) 11
4.2.3 Safety population 11
4.3 Estimands 11
4.3.1 Treatment policy estimand 11
4.3.2 Per Protocol analysis 12

5. Trial Population 13
5.1 Screening data 13
5.2 Eligibility 13
5.3 Recruitment 14
5.4 Cluster characteristics 14
5.5 Baseline patient characteristics 14
5.6 Adherence and protocol deviations 15
5.7 Withdrawal/follow-up 16

6. Analysis 17
6.1 Outcome definitions 17
6.1.1 Primary outcome 17
6.1.2 Other outcomes of interest 17
6.1.3 Safety outcomes 17
6.2 Analysis methods 18
6.2.1 Primary analysis 18
6.2.2 Secondary analyses 18
6.2.3 Sensitivity analyses 19
6.2.4 Subgroup analyses 19
6.2.5 Additional analyses 19
6.2.6 Assessment of statistical assumptions 19
6.3 Interim analyses 19

6.4 Missing data 19
6.5 Safety evaluation 20
6.6 Statistical software 20
6.7 Quality control 20

7. Changes from the protocol 21

8. References 22

1. Introduction

1.1 Background and rationale/

Common mental disorders are highly prevalent among people living with HIV. Left untreated, common mental disorders cause substantial disability and undermine individuals' ability to adhere to antiretroviral therapy (ART), leading to poor HIV treatment outcomes. Management of mental disorders is a promising strategy to improve both mental health and HIV treatment outcomes among people on antiretroviral therapy who also suffer from common mental disorders. A recent cluster-randomized controlled trial from Harare, Zimbabwe showed that problem-solving therapy delivered by lay health workers effectively reduced symptoms of common mental disorders, but the effect of the intervention on HIV treatment outcomes and its effectiveness in the rural setting has not been studied. We aim to examine the effect of problem-solving therapy delivered by lay health workers on HIV treatment outcomes in rural Zimbabwe.

1.2 Objectives

Primary objective: To assess the effect of problem-solving therapy on HIV treatment outcomes.

Secondary objectives: To assess the effectiveness of problem-solving therapy on symptoms of common mental disorders among rural HIV-infected populations and to assess the prevalence of common-mental disorders among rural HIV-infected population on ART.

2. Study methods

2.1 Trial design

The study is designed as a cluster-randomized, controlled, parallel, two-arm multicenter trial with 1:1 allocation ratio.

2.2 Randomization

Health facilities will be assigned in a 1:1 ratio to the intervention or the control group using a computer-generated, stratified blocked randomization. Randomization will be stratified by clinic size (<280; 280-1000, >1000 active ART patients). To avoid imbalance in the size of the groups, we will use blocked randomization within each stratum, with blocks consisting of two health facilities. The unit of random allocation will be the health facility and not the individual participant. This will minimize the risk of contamination between study arms. Randomization of health facilities was done by a statistician who is not part of the study team at CTU Bern, Switzerland. Randomization will be done prior to initiation of the recruitment phase.

2.3 Sample size

We will recruit 480 participants (16 clusters with 30 participants each). See section 11.2 in the study protocol (version 4) for determination of sample size.

2.4 Framework

Trial to show superiority of problem-solving therapy (intervention) over enhanced standard of care (control).

2.5 Statistical interim analyses and stopping guidance

There is no interim analysis planned.

2.6 Timing of final analysis

Final analysis will take place once all follow-up activities are completed, all data is entered in the database, and all data is statistically validated and cleaned in the database. Before starting the analysis the database will be locked and a final export of study data will be done.

2.7 Timing of outcome assessments

Study outcomes are assessed at 3, 6, 9, and 12 months after recruitment.

2.8 Blinding

Treatment assignment is unblinded to participants, lay health workers, health care providers, evaluators, data managers, and trial statistician.

The senior statistician authoring the SAP is blinded while writing the SAP, during the whole conduct of the study and final analysis. The senior statistician is only unblinded before quality control (i.e. double programming) of the primary analysis.

3. Data management

3.1 Data export

The CRFs in this trial are implemented electronically using a dedicated electronic data capturing (EDC) system (REDCap, https://www.project-redcap.org/). All data entered in the eCRFs are stored on a Linux server in a dedicated mySQL database. Responsibility for hosting the EDC system and the database lies with CTU Bern.

Data on medication adherence is collected with the Medication Event Monitoring System ([MEMS], AARDEX, Sion, Switzerland). Adherence is measured with patient-held electronic pill bottles (MEMS caps) that record the dosing history of a patient (i.e. the time of each bottle opening). Research assistants download data recorded on MEMS caps at 3-monthly follow-up visits using Android tablets and synchronize the tablets with the medAmigo web platform. MedAmigo is an online web-solution to retrieve and transfer dosing history data from MEMS caps through the internet to dedicated servers. The dosing history data are stored on centralized, secured servers and regular backups are scheduled. Responsibility for hosting the medAmigo system and the database lies with the manufacturer of the MEMS (AARDEX, Sion, Switzerland). At final analyses, data files will be extracted from the database and imported into a statistical software package according to the SOP for data preparation and programming[i].

3.2 Data validation

First line data validation is performed by the online eCRF system at real-time as defined in the data dictionary. Second line data validation and cleaning will be performed after completion of data entry but before database lock according to the SOP for data validation[ii].

Data checks are described in the central data-monitoring plan.

3.3 Data preparation

3.3.1 Adherence data (MEMS data)

Participants' 1-monthly (30-days) mean adherence score is calculated as the percentage of doses correctly taken per interval. For regimens that have to be taken once daily, a dose is considered as taken if the participant opens the electronic pill bottle within -8 to +16 hours of the designated dosing time. For regimens that have to be taken twice daily, a dose was considered as taken if the participant opens the electronic pill bottle within -4 to +8 hours of the designated dosing time. The designated dosing time is specific to each patient and recorded in the eCRF.

3.3.2 SSQ-14

The SSQ-14 is a dichotomous 14-item questionnaire. Each item where the answer is yes is scored as 1, providing a score ranging from 0 to 14.

3.3.3 PHQ-9

Nine items, each of which is scored 0 to 3 on a Likert scale, providing a severity score ranging from 0 to 27.

3.3.4 Viral load suppression

Will be assessed as dichotomous considering <1000 copies per milliliter as suppressed.

3.3.5 Safety outcomes

Psychiatric hospitalization and suicide attempts are reported in variables rf_1 and rf_2, which are assessed at each visit.

Suicides are assessed based on the unscheduled serious adverse event form, using the variables sae_type, sae_description, sae_type_fup1, sae_description_fup1, sae_type_fup2, and sae_description_fup2. Sae_description are free text fields and will be assessed by an appropriately trained person.

3.3.6 Data sharing

Data sharing is described in the data management plan.

4. Statistical principles

4.1 Confidence intervals and *P* values

All applicable statistical tests will be 2-sided and will be performed using a 5% significance level. 95% confidence intervals will be reported.

4.2 Analysis populations

4.2.1 Full analysis set (FAS)

The full analysis set (FAS) will include all eligible participants who consented to participate in the study and attended the baseline visit. Following the intent-to-treat principle, subjects will be analyzed according to the treatment which was assigned to the site they are enrolled at.

Table 1: Violation of eligibility criteria

Protocol deviation	eCRF visit	Variable	Variable type	Violation
Violation of inclusion or exclusion criteria				
Age >18 years	Recruitment	es_2	Continuous: years	es_2<18
On first-line antiretroviral therapy for at least 6 months	Recruitment	es_4 and<br>es_14 – es_3_date and<br>es_18	Dichotomous: multiple choice<br>Continuous: days<br>Dichotomous: multiple choice	es_4 = 2, -8, -9<br>es_14 – es_3_date < 180<br>es_18 = 7, -7
Resident in Bikita District	Recruitment	es_6	Dichotomous: multiple choice	es_6 = 2, -8, -9
Speak and understand English or Shona	Recruitment	es_8	Dichotomous: multiple choice	es_6 = 0
Able to comprehend the information on the study	Recruitment	es_21	Dichotomous: multiple choice	es_21 = 0
Screened positive for CMDs (Shona Symptoms Questionnaire ≥9)	Recruitment	ssq14_1_score	Continuous: score	ssq14_1_score < 9
Provided written informed consent (consent by thumbprint) to participate study	Recruitment	es_21	Dichotomous: multiple choice	es_21 = 0
Current psychosis / cognitive impairment	Recruitment	ssq14_1_5 and<br>es_27	Dichotomous: multiple choice<br>Dichotomous: multiple choice	ssq14_1_5 = 1<br>es_27 = 1, -7
Clinical AIDS (WHO clinical stage 4)	Recruitment	es_23	Dichotomous: multiple choice	es_23 = 4, -7
Known pregnancy or ≤3 months postpartum	Recruitment	es_5	Dichotomous: multiple choice	es_5 = 1, -8, -9

4.2.2 Per-protocol (PP)

Per-protocol population consists of all subjects in the FAS who do not have any protocol deviations that could confound the interpretation of analyses conducted on the FAS. The following are common major protocol deviations:

- Not receiving allocated intervention
- Completely missing primary outcome data (2-6 months)

Table 2: Derivation of protocol deviations.

Protocol deviation	eCRF visit	Variable	Variable type	Derivation
Not receiving allocated intervention				
Attending less than four friendship bench individual counselling sessions for participants in the intervention group.	Friendship Bench 1 - 4	fb_date	Continuous: date	fb_date = less than 4 completed
Attending no nurse-led mental health counselling for participants in the control and intervention group.	Nurse-led mental health counselling	nlc_yn	Dichotomous: multiple choice	nlc_yn = 2, -8 or -7
Receiving un-allocated intervention				
Attending one or more friendship bench individual or group counselling sessions for participants in the control group.	Any Friendship Bench Visit	fb_date	Continuous: date	fb_date = any completed
Completely missing primary outcome data (percentage adherence at month 2 to month 6).	MEMS data	adh2 – adh6	Continuous	adh2-adh6 all missing

4.2.3 Safety population

The safety population consists of all subjects in the FAS. Subjects will be analyzed according to the treatment they actually received.

4.3 Estimands

The ICH E9 (R1) addendum on estimands and sensitivity analyses defines different treatment estimators that are of interest in clinical trials (EMA 2017). An estimand is the target of estimation to address the scientific question of interest posed by the trial objective. Attributes of an estimand include the population of interest, the outcome of interest, the specification of how intercurrent events such as crossovers and non-compliance are reflected in the scientific question of interest, and the population-level summary for the outcome.

4.3.1 Treatment policy estimand

The estimand that addresses the main objective of this trial is based on the treatment policy strategy. The value for the outcome of interest is used regardless of whether or not the intervention was performed

and/or recommendations war applied. The primary intention-to-treat analysis of the primary as well as secondary outcomes will be based on this estimand using the FAS.

- Primary outcome of interest: Mean adherence between 2-6 months
- Patient-set of interest: FAS
- Handling of intercurrent events: Outcome data will be used anyway; if missing, outcome data will be imputed
- Population-level summary measure of outcome: Difference in mean adherence between 2-6 months

4.3.2 Per Protocol analysis

We will perform a per-protocol analysis. Subjects with major protocol deviations will be excluded from this analysis.

5. Trial Population

5.1 Screening data

We will draw a flow diagram following the CONSORT extension for Cluster Trials 2012 (http://www.consort-statement.org/extensions/overview/cluster-trials). In addition to the number, average cluster size, and variance of cluster size, we will report number and percentage of patients.

Flowchart 1: CONSORT

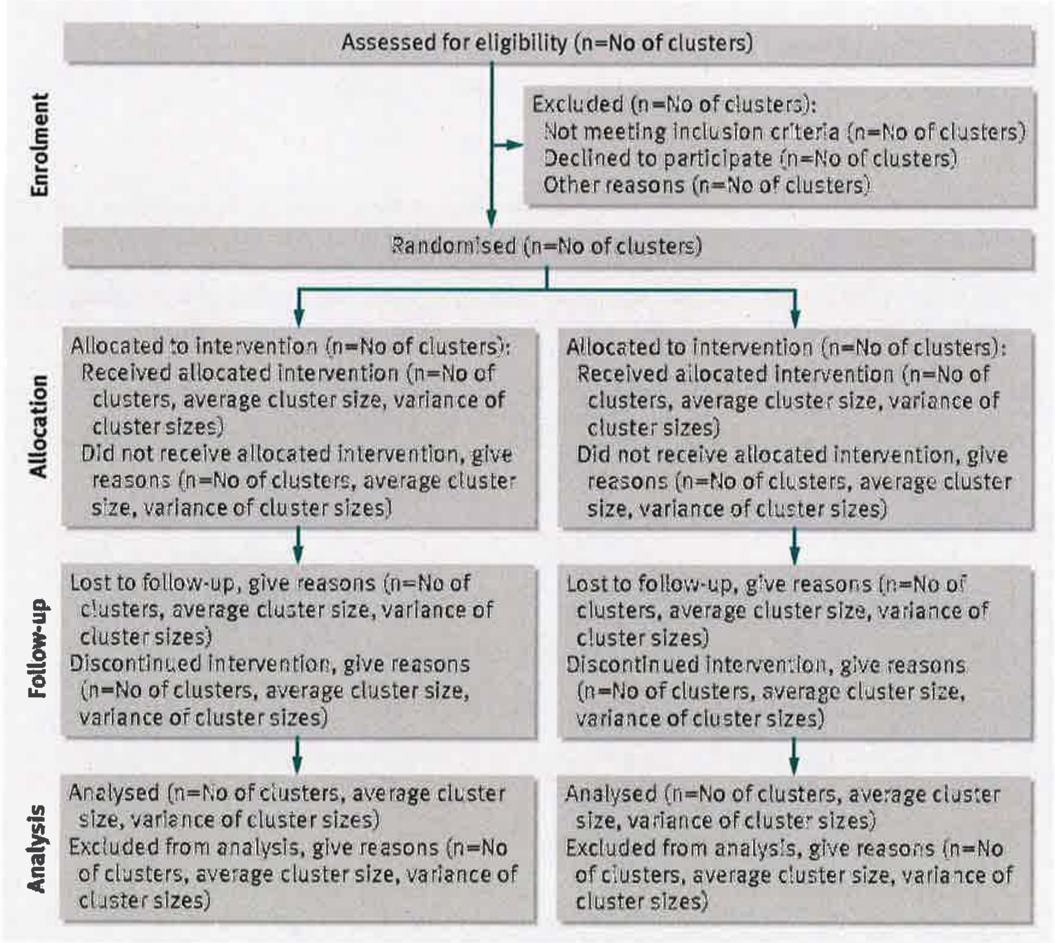

5.2 Eligibility

Inclusion criteria

- Age >18 years
- On first-line antiretroviral therapy for at least 6 months
- Resident in Bikita District
- Speak and understand English or Shona
- Able to comprehend the information on the study

- Screened positive for CMDs (Shona Symptoms Questionnaire ≥9)
- Provided written informed consent (consent by thumbprint) to participate study

Exclusion criteria

- Current psychosis / cognitive impairment
- Clinical AIDS (WHO clinical stage 4)
- Known pregnancy or ≤3 months postpartum

5.3 Recruitment

Information is included in the CONSORT flow diagram (flowchart 1).

5.4 Cluster characteristics

Cluster characteristics at baseline will be presented in a table, stratified by treatment arm, as number and percentage or mean and standard deviation for categorical and normally distributed continuous variables, respectively. For data severely deviating from a normal distribution, we will present median and interquartile ranges. Groups will not be compared statistically because any significant difference can be explained by the play of chance if the randomization was performed properly.

Table 2: Cluster characteristics

Description	Variable	Type
Type of clinic	cluster_type	Categorical: multiple choice
Clinic size (active ART patients)	cluster_size	Categorical: multiple choice
Average travel time to reach facility	b_hs_4	Continuous: Traveling hours
Average travel cost to reach facility	b_hs_7	Continuous: Dollars
Average duration of visit at facility	b_hs_6	Continuous: Hours at clinic

5.5 Baseline patient characteristics

The patient characteristics of the FAS at baseline will be presented in a table, stratified by treatment arm, as number and percentage or mean and standard deviation for categorical and normally distributed continuous variables, respectively. For data severely deviating from a normal distribution, we will present median and interquartile ranges. We will show p-values for differences between the two groups because cluster-randomized trials lack the balancing properties of classical randomized trails. Intervention groups will be compared by the t-test for continuous data or by the non-parametric Wilcoxon test when normality assumption is not satisfied. For categorical data, Fisher's exact test will be used if expected frequencies are lower than 5 in any cell, else the chi-squared test will be applied.

Table 3: Patient characteristics table.

Description	Variable	Type
Age	es_14	Continuous: dates

Description	Variable	Type
Gender	gender	Binary: male, female
Marital status	b_marital	Categorical: married or living together, widowed, divorced, or separated, single.
Education	b_school, b_years	Categorical: None or primary, at least secondary
Religion	b_religion	Categorical: Christian or other
SSQ-14 score	ssq14_1_score	Continuous: score
PHQ 9 Score	phq9_1_score	Continuous: score
Depression	Phq9_1_score	Categorical: PHI 9 <11 or ≥11
WHO clinical stage	es_23	Dichotomous: stage 1 - 4
Viral load suppression (<1000 cop-ies/mL)	lab_vls_bl and lab_vl_bl	Dicotomous: Viral load not detectable or below 1000 copies/mL)
CD4 cell count	lab_cd4	Continuous: cell count
ART regimen	es_18	Dichotomous: Efavirenz or other
Comprehensive HIV knowledge	b_hiv_3 – b_hiv_7	Categorical: all five HIV knowledge questions correctly answered? Yes/no
Comprehensive ART knowledge	b_art_1 – b_art_4	Categorical: all four ART knowledge questions correctly answered? Yes/no
HIV status disclosure to family or a friend	b_hiv_1	Categorical: yes, no, refused/don't know
Household Food Insecurity scale (HFIS) score	b_food_1 – b_food_14	Continuous: score
Medical Outcomes Study Social Support Survey (MOS-SS) score	b_social_1 – b_social_8	Continuous: score
Alcohol use: AUDIT-C score	b_alc_1 – b_alc_3	Continuous: score
Patient–Provider Relationship Scale (PPRS) score	b_hs_1 – b_hs_9	Continuous: score
General health perception	Medical Outcomes Study Short Form Survey Instrument (MOS SF-36) item	Categorical:
Perceived effectiveness of ART	MOS SF-36 item: b_hs_9	Categorical:
Attending support group for PLHIV	b_hs_4	Categorical: yes, no, refused/don't know
Health care decision making	b_gender_1	Categorical: participant or other

5.6 Adherence and protocol deviations

Adherence to the interventions and protocol deviations will be assessed based on the extent of exposure to the assigned intervention.

Adherence to the interventions will be defined as:

- Intervention arm: receiving at least four friendship bench individual counselling sessions and nurse-led mental health counselling
- Control arm: receiving nurse-led mental health counselling

Major protocol deviations will be defined as:

- Control arm: not receiving nurse-led mental health counselling or receiving one or more friendship bench individual counselling sessions or friendship bench group support
- Intervention arm: receiving less than four friendship bench individual counselling sessions or not receiving nurse-led mental health counselling

Description of how adherence to the intervention will be presented:

- N_{int} allocated to intervention
- N_{int} received nurse-led mental health counselling & at least four friendship bench individual counselling sessions
- N_{int} did not receive received nurse-led mental health counselling or did not receive at least four friendship bench individual counselling sessions (i.e. premature end of study)
- N_{int} received nurse-led mental health counselling
- N_{int} received individual counselling session for each of the six visits
- N_{int} received friendship bench group counselling
- Median (IQR) number of friendship bench group counselling visits attended

- N_{cont} allocated to control
- N_{cont} received nurse-led mental health counselling
- N_{cont} did not receive nurse-led mental health counselling (premature end of study)

5.7 Withdrawal/follow-up

All withdrawals and losses to follow-up will be listed with the time points and reasons (if available).

6. Analysis

6.1 Outcome definitions

6.1.1 Primary outcome

The primary outcome is the mean adherence between 2-6 months (i.e. start of month 2 until end of month 6, total of 5 months (1 month = 30 days)).

We will calculate participants' 1-monthly mean adherence scores as the percentage of doses taken per interval. For regimens that have to be taken once daily, a dose is considered as taken if the participant opens the electronic pill bottle within -8 to +16 hours of the reported usual dosing time (es_24). For regimens that have to be taken twice daily, a dose was considered as taken if the participant opens the electronic pill bottle within -4 to +8 hours of the reported usual dosing time for the morning dose (es_25) or evening dose (es_26), respectively. We will consider 1-monthly mean adherence scores of <10% (i.e. <3 bottle openings) as missing data assuming that participants with very few openings did not consistently use their MEMS caps.

Secondary outcomes

- Mean adherence between 1-12 months (at each month, i.e. for each 1-monthly interval)
- Change from baseline in SSQ-14 score at 3 months (i.e. day 90 ± 45 days), 6 months (i.e. day 180 ± 45 days), 9 months (i.e. day 270 ± 45 days) and 12 months (i.e. day 360 - 45 /+ 60 days).
- Change from baseline in PHQ-9 score at 3 months (i.e. day 90 ± 45 days), 6 months (i.e. day 180 ± 45 days), 9 months (i.e. day 270 ± 45 days) and 12 months (i.e. day 360 - 45 + 60 days), the PHQ score will be calculated based on the test manual.
- Viral load suppression (<1000 copies per milliliter) at 6 months (i.e. day 180 ± 90 days) and 12 months (i.e. day 360 ± 90 days), categorical (yes, no, invalid or missing).

6.1.2 Other outcomes of interest

- Common-mental disorders (SSQ-14 ≥9) at 3 months (i.e. day 90 ±45 days), 6 months (i.e. day 180 ±45 days), 9 months (i.e. day 270 ±45 days) and 12 months (i.e. day 360 – 45 /+ 60 days).
- Depression (PHQ-9 ≥11) at 3 months (i.e. day 90 ±45 days), 6 months (i.e. day 180 ±45 days), 9 months (i.e. day 270 ±45 days) and 12 months (i.e. day 360 – 45 /+ 60 days).

6.1.3 Safety outcomes

- Psychiatric hospitalization up to 12 months (i.e. day 360 + 30 days)
- Suicide attempt up to 12 months (i.e. day 360+ 30 days)
- Suicide up to 12 months (i.e. day 360+ 30 days)

Table 4: Derivation of primary and secondary outcomes.

Outcome	eCRF sheet	Variable	Outcome type
**Primary**: Mean adherence between 2 – 6 months	Continuous data extracted from medAmigo	adh2 – adh6	Continuous
**Secondary**: Mean adherence at month 1 - 12	Continuous data extracted from medAmigo	adh1 – adh12	Continuous

**Secondary**: Change in SSQ-14 and PHQ-9 scores	Baseline: ssq14 and phq9<br>Follow-ups: ssq14 and phq9	ssq14_1_score<br>phq9_1_score<br>ssq14_1_score_v2<br>phq9_1_score_v2	Continuous
**Secondary**: Proportion of virologic suppressed	Follow-up: detectable viral load (or below 1000 copies / ml) or not	lab_vls_6<br>lab_vls_12	Dichotomous
**Other**: Common-mental disorders (SSQ-14)	Follow-ups: ssq14	ssq14_1_score_v2	Dichotomous: ≥9 or <9
**Other:** Depression (PHQ-9 ≥11)	Follow-ups: phq9	phq9_1_score_v2	Dichotomous: ≥11 or <11
**Safety:** Psychiatric hospitalisation	Any visit after recruitment	rf_1	Dichotomous: yes
**Safety:** Suicide attempt	Any visit after recruitment	rf_2	Dichotomous: yes
**Safety:** Suicide	Any visit after recruitment	sae_type and sae_description	Dichotomous: death and described as suicide

6.2 Analysis methods

Analyses will be done at the Institute of Social and Preventive Medicine with support of CTU Bern.

6.2.1 Primary analysis

The primary analysis will be based on the intention-to-treat principle using the FAS. Missing data will be handled according to section 6.4.

We will depict the mean adherence at baseline and each month of follow-up for both groups in a graph showing the point estimate as well as a 95% confidence interval. We will use linear mixed effect models to assess the difference in mean adherence. Models include a random intercept and slope on the participant-level to account for correlation of measurements within participants, a random intercept on the cluster-level to account for clustering of individuals in health facilities, an indicator for treatment assignment, an indicator for each month of analysis time, and interactions of treatment assignment and each month of analysis time. The primary outcome (mean adherence between 2 and 6 months) as well as secondary adherence outcomes (mean adherence at each month) will be assessed from these models based on contrasts.

We will also use linear mixed effect models to assess the difference (95% confidence interval) in change from baseline in SSQ-14 and PHQ-9 score. Models include a random intercept and slope for participants, a random intercept on the cluster level, an indicator for treatment assignment, the respective baseline value of either SSQ-14 or PHQ-9, an indicator for month of analysis time, and interactions of treatment assignment and analysis time.

We will use logistic mixed effect models to assess the difference in the proportion of participants with viral load suppression, with common-mental disorders (SSQ-14 ≥9), and with depression (PHQ-9 ≥11) at 6 and 12 months. Models include a random intercept on the cluster level, and an indicator for treatment assignment. We will present results as OR with 95% confidence interval.)

6.2.2 Secondary analyses

In a per-protocol analysis, we will evaluate outcomes as described in 6.2.1 using the per-protocol patient population.

Because cluster-randomization may lack the excellent balancing properties of individual-level randomization, we will adjust models in a secondary intention-to-treat analysis using the FAS. We will adjust models of adherence outcome data for the size of the clinic, age, gender, type of ART regimen, and CD4 at baseline. Other models will be controlled for the size of the clinic, age, and gender.

Moreover, we will analyze all outcomes on the cluster level using aggregated data. Continuous data will be averaged on the cluster level. For binary data, the proportion will be calculated for each cluster. Averaged data will be compared between groups using a non-parametric Wilcoxon rank-sum test.

6.2.3 Sensitivity analyses

In a sensitivity analysis, we will analyze primary and secondary adherence outcomes as count data based on the FAS. We will use a generalized mixed-effects linear model with a negative binomial distribution, a log-link, a random intercept on the cluster level, an indicator for treatment assignment, and an offset for exposure time. The exposure time will be the number of days with available adherence data (i.e. a maximum of 5 months for the primary outcome and a maximum of 30 days for secondary outcomes).

We will consider a further sensitivity analysis using the FAS if there are pronounced imbalances in patient- and cluster-level baseline characteristics that were not already considered in the secondary analysis, by further adjusting models for these imbalanced variables.

If multiple imputation is employed in the primary analysis, we will additionally perform a complete case analysis based on the FAS.

To see whether the adherence is sensible to the time window (-8 to +16 hours), we will do the analysis of the outcomes addressing adherence by considering a dose as taken if the participant opens the electronic pill bottle within -6 to +6 hours of the reported usual dosing time (es_24 or es_25 and es_26).

6.2.4 Subgroup analyses

Any planned subgroup analyses for each outcome including how subgroups are defined

Table 5: Derivation of subgroups

Subgroup	eCRF visit	Variable	Categorization
Disease severity (PHQ-9 ≥11 vs <11)	Follow-ups: phq9	phq9_1_score_v2	Dichotomous: ≥11 or <11
Men vs. women	Recruitment	gender	Binary: male, female
Young vs. old (median)	Recruitment	es_2	Continuous: years

6.2.5 Additional analyses

We will evaluate the intra-class correlation (ICC) of the primary and all secondary outcomes.

6.2.6 Assessment of statistical assumptions

Model assumptions for continuous data will be checked visually using plots of residuals (residuals vs fitted values, QQ-plot). If model assumptions are violated, transformation of the outcome (e.g. log-transformation), more robust methods (e.g. robust standard errors or robust regression) or analysis of aggregated data on the cluster level will be considered.

6.3 Interim analyses

There is no interim analysis planned.

6.4 Missing data

If there are patients with completely missing outcome data during the whole follow-up, we will use multiple imputation assuming missing data to be missing at random. If there is missing data at some but

available data at other time points, we will use mixed-effects repeated measures models instead, which account for partially missing data.

If multiple imputation is indicated, each outcome will be imputed separately. We will consider all baseline variables (see Table 5.5 with patient characteristics), outcome measures at all time-points as well as an indicator for the treatment and an indicator for the cluster as predictors in the imputation models. Variables with more than 50% missing values will not be used for the imputation model. Binary variables with a frequency of less than 5% in one category will be omitted, levels of categorical variables with a frequency of less than 5% in one category will be combined with another level in a sensible way. Continuous variables will be log-transformed if it improves normality (checked by Shapiro-Wilks test and QQ plots). We will use multiple imputation by chained equation. Predictive mean matching will be used for continuous and ordinal variables, logistic regression for binary and multinomial regression for categorical variables. In total, fifty imputed data sets will be generated, which will be analyzed using Rubin's rules (Rubin 1987).

6.5 Safety evaluation

Safety outcomes will be summarized descriptively, showing the number and proportion with a 95% Wilson confidence interval separate for both groups.

6.6 Statistical software

All statistical analyses will be done in Stata 15.0 (or a subsequent version).

6.7 Quality control

A senior statistician from CTU Bern will reproduce the primary intention-to-treat analysis of the primary and secondary outcomes. Analyses will be based on aggregated 1-monthly MEMS adherence data and clinical data exported from the database system, i.e. multiple imputation will also be reproduced. If results deviate from the original analysis, the reason for the difference will be determined and a consensus must be reached.

All other analyses will be quality-controlled by the senior statistician performing a review of the statistical report.

7. Changes from the protocol

The SAP is consistent with principle features of the statistical methods described in the protocol. Any deviation from the protocol is detailed hereunder with reason.

Table 6: Changes from protocol

Header	Change	Reason
5.2 Secondary outcomes	The outcomes 'change from baseline in SSQ-14 score and PHQ-9 score at 3 months and 9 months' were added.	These questionnaires were also collected at month 3 and 9 (besides 6 and 12) and will therefore also be evaluated.
5.2 Secondary outcomes	The outcome 'change from baseline in proportion of participants with a viral load suppression (<20 copies per milliliter) at 6 months and 12 months' was formulated at the patient level. Moreover the threshold was adapted to <1000 copies.	The detection limit is 1000 copies and collected accordingly in the eCRF.
11.3.2 Primary analysis	We deleted the linear mixed effect model for the change from baseline in the proportion of participants with viral load suppression at 12 month.	This population-level outcome cannot be analyzed at the patient level.
11.3.4 Safety analysis	We will calculate the number and proportion of patients with safety outcomes, not incidence rates and rate ratios.	We only expect few safety outcomes on the patient level and overall and therefore decided to present simple descriptive statistics.

8. References

European Medicines Agency, Committee for Human Medicinal Products; Draft ICH E9 (R1) addendum on estimands and sensitivity analysis in clinical trials to the guideline on statistical principles for clinical trials, step 2b - Revision 1. 30 August 2017.

SOP Data preparation and data analysis, CS_STA_SOP_05, version 04, 11.09.2018

SOP Statistical data validation, CS_STA_SOP_02, version 03, 11.09.2018

StataCorp. 2017. Stata Statistical Software: Release 15. College Station, TX: StataCorp LLC

Rubin, DB (1987). Multiple Imputation for Nonresponse in Surveys. New York: J Wiley & Sons. 1987
