# Supplementary material for: Effect of the Friendship Bench Intervention on Antiretroviral Therapy Outcomes and Mental Health Symptoms in Rural Zimbabwe: A Cluster Randomized Trial
Source: JAMA Netw Open. 2023 Jul 13;6(7):e2323205. doi: 10.1001/jamanetworkopen.2023.23205 (PMC10346120; doi:10.1001/jamanetworkopen.2023.23205)
Supplement: Supplement 2. — eMethods. Additional Information on Standard Care, Randomization, Adherence Measures, and Statistical Analyses eTable 1. Effect of the Friendship Bench Intervention on Adherence, Viral Load, and Mental Health: Results From Prespecified and Post Hoc Sensitivity Intention-to-Treat Analyses eTable 2. Effect of the Friendship Bench Intervention on Adherence, Viral Load, and Mental Health: Results From Prespecified and Post Hoc Sensitivity Per-Protocol Analyses eTable 3. Number of Participants With Safety-Relevant Outcomes by Trial Group eFigure. Forest Plot of Available Evidence of the Effect of the Friendship Bench Intervention on SSQ-14 Scores, PHQ-9 Scores, and Viral Suppression From 3 Trials [file jamanetwopen-e2323205-s002.pdf]

Supplemental Online Content

Haas AD, Kunzekwenyika C, Manzero J, et al; Friendship Bench ART trial group. Effect of the Friendship Bench intervention on antiretroviral therapy outcomes and mental health symptoms in rural Zimbabwe: a cluster randomized trial. *JAMA Netw Open*. 2023;6(7):e2323205. doi:10.1001/jamanetworkopen.2023.23205

eMethods. Additional Information on Standard Care, Randomization, Adherence Measures, and Statistical Analyses

eTable 1. Effect of the Friendship Bench Intervention on Adherence, Viral Load, and Mental Health: Results From Prespecified and Post Hoc Sensitivity Intention-to-Treat Analyses

eTable 2. Effect of the Friendship Bench Intervention on Adherence, Viral Load, and Mental Health: Results From Prespecified and Post Hoc Sensitivity Per-Protocol Analyses

eTable 3. Number of Participants With Safety-Relevant Outcomes by Trial Group

eFigure. Forest Plot of Available Evidence of the Effect of the Friendship Bench Intervention on SSQ-14 Scores, PHQ-9 Scores, and Viral Suppression From 3 Trials

This supplemental material has been provided by the authors to give readers additional information about their work.

© 2023 Haas AD et al. *JAMA Network Open.*

eMethods. Additional Information on Standard Care, Randomization, Adherence Measures, and Statistical Analyses

Enhanced adherence counseling guidelines

Antiretroviral therapy providers who were not part of the study team conducted enhanced adherence counselling sessions. Counselling sessions were conducted according to the Operational and Service Delivery Manual for the Prevention, Care, and Treatment of HIV in Zimbabwe.[1] The first session consists of nine steps: viral load education review, a discussion of the patient's reason for a high viral load, review of the patient's medication dosing schedule, planning for the storing of medications, developing the patient's motivation card, a discussion of the patient's support system, planning for substance use, addressing issues related to getting to clinic appointments, and finally planning the next steps.[1] The second session is held one month after the first session and builds on the content discussed in the first session. The session has the following objectives: identifying any difficulties addressing issues with the plan, engaging in discussions on how to learn from mistakes, reviewing and attending to any possible referrals that the patient may have gone to, discussing how to handle travel while on medication, and finally planning the next steps.[1] Additional counseling sessions can be planned as needed.

Randomization

Randomization was stratified by the size of the health facility (<280, 280-1000, >1000 retained ART patients). We used block randomization within each stratum, with blocks consisting of two health facilities, to balance the size of the groups. Randomization was done by a statistician who was not part of the study team.

Standard operating procedures for follow-up and referral of suicidal or psychotic participants

We used a red flag system to alert us to the possible occurrence of self-harm, suicide, or psychosis. A red flag was present when a participant had responded "yes" to item 5 (hallucinations) or item 11 (suicidality) of the SSQ-14. Participants were assessed for a red flag at every visit by the research assistant, during nurse-led counseling by the nurse, and during the intervention visits by lay health workers. Identified red flags were referred to the nurse in charge at the clinic, who assessed and referred to the medical officer at the provincial psychiatric unit if needed for further management.

Adherence measures

We used electronic pill caps (Medication Event Monitoring System [MEMS], AARDEX, Sion, Switzerland) to measure adherence during follow-up. We considered a dose missing if no bottle opening was recorded on a particular day. This definition deviated from the statistical analysis plan, which defined a dose as missing if no bottle opening was recorded within a time window of 8 hours before and 16 hours after the designated dosing time. This deviation was necessary because, in some clusters, for about one-third of participants, the reported designated dosing time was incorrectly recorded as it deviated by 6-12 hours from the observed typical dosing time.

We assessed self-reported baseline adherence using a 30-day recall based on the following item from the AIDS Indicator Survey.[2]

“People sometimes forget to take all their ARVs every day. In the last 30 days, how many days have you missed taking any of your ARV pills?”

Statistical models

© 2023 Haas AD et al. *JAMA Network Open.*

We used linear mixed-effects models to assess the difference in mean adherence. Models included a random intercept and slope at the participant level to account for the correlation of measurements within participants. A random intercept accounted for the clustering of individuals in health facilities, and indicators defined treatment assignment, the month of analysis time, and interactions between the two. We estimated marginal odds ratios for the difference in the proportion of participants with viral suppression at 6 and 12 months using logistic mixed-effect models. We conducted prespecified adjusted analyses of mean adherence and viral suppression, controlling for facility size, age, and sex. In post hoc sensitivity analyses, we adjusted for facility size, age, sex, self-reported baseline adherence, baseline SSQ-14 score, ART regimen, PHQ-9 score, WHO clinical stage, CD4 cell count, viral suppression, audit score, MOS-SS score, and travel cost. We also assessed the difference in change from baseline in SSQ-14 and PHQ-9 scores.

We used logistic mixed-effect models to assess the difference in the proportion of participants with common-mental disorders (SSQ-14 >9) and with depression (PHQ-9>11) at 3, 6, 9, and 12 months. Models included a random intercept at the cluster level and an indicator for treatment assignment. We controlled for facility size, age, and sex in prespecified adjusted analyses. We did a post hoc sensitivity analysis adjusting for facility size, age, sex, baseline SSQ-14 or PHQ-9 score, WHO clinical stage, CD4 cell count, viral suppression, audit score, and MOS-SS score. We repeated analyses of primary and secondary outcomes using a per-protocol analysis, excluding participants who did not receive the allocated intervention and those with missing adherence data between months 2 to 6.

Multiple imputation models

Missing data were imputed using multiple imputation by chained equations.[3] Analyses were run on 30 imputed datasets, and results combined using Rubin's rule. We imputed the four outcomes separately. For adherence, imputation models included monthly mean adherence scores for all time points, an indicator for study facility, age, sex (male or female), marital status (married/living together, or single/widowed/divorced/separated), education (primary or secondary), the baseline SSQ-14 and PHQ-9 scores, the baseline CD4 cell count, viral suppression, WHO clinical stage, ART regimen (NNRTI-based, PI-/II-based), the self-reported baseline adherence score, the time since ART initiation, the HFIS, MOS-SS, and AUDIT-C scores, comprehensive ART knowledge (yes or no) and transportation cost to the clinic (<2 USD, 2-5 USD, >5USD).

For viral suppression, imputation models included viral suppression at all time points, an indicator for treatment assignment, age, sex (male or female), marital status (married/living together, or single/widowed/divorced/separated), education (primary or secondary), the baseline SSQ-14 and PHQ-9 scores, the baseline CD4 cell count, WHO clinical stage, the self-reported baseline adherence score, the ART regimen (NNRTI-based, PI-/II-based), the MOS-SS score, and the AUDIT-C score.

Imputation models for SSQ-14 scores included SSQ-14 scores at all time points, an indicator for study facility, age, sex (male or female), marital status (married/living together, or single/widowed/divorced/separated), education (primary or secondary), the baseline PHQ-9 scores, the baseline CD4 cell count, viral suppression, WHO clinical stage, the self-reported baseline adherence score, the time since ART initiation, the HFIS, MOS-SS, and AUDIT-C scores.

Imputation models for PHQ-9 scores included PHQ-9 scores at all time points, an indicator for study facility, age, sex (male or female), marital status (married/living together, or single/widowed/divorced/separated), education (primary or secondary), the baseline SSQ-14 scores, the baseline CD4 cell count, viral suppression, WHO clinical stage, the self-reported baseline adherence score, the time since ART initiation, the HFIS, MOS-SS, and AUDIT-C scores.

© 2023 Haas AD et al. *JAMA Network Open.*

eReferences

1. AIDS & TB Programme Ministry of Health and Child Care Zimbabwe. *Operational and Service Delivery Manual for the Prevention, Care and Treatment of HIV in Zimbabwe*.; 2017. https://differentiatedservicedelivery.org/Portals/0/adam/Content/m2an155byU6RIoHeF4e4FQ/File/MSF Zim OSDM web revised.pdf

2. AIS. AIDS Indicator Survey (AIS). Published 2017. Accessed January 21, 2021. https://dhsprogram.com/What-We-Do/Survey-Types/AIS.cfm

3. Rubin DBD. *Multiple Imputation for Nonresponse in Surveys*. (Rubin DB, ed.). John Wiley & Sons, Inc.; 1987. doi:10.1002/9780470316696

© 2023 Haas AD et al. *JAMA Network Open.*

eTable 1. Effect of the Friendship Bench Intervention on Adherence, Viral Load, and Mental Health: Results From Prespecified and Post Hoc Sensitivity Intention-to-Treat Analyses

	Prespecified analyses				Sensitivity analyses (post hoc)	
**Outcome**	**Unadjusted mean difference (95% CI)**	**p-value**	**Adjusted mean difference (95% CI)**	**p-value**	**Adjusted mean difference (95% CI)**	**p-value**
MEMS adherence						
Months 2-6	1.93 (-1.20 to 5.06)	0.2276	1.79 (-1.71 to 5.29)	0.3160	2.05 (-1.91 to 6.01)	0.3098
Months 1-12	0.79 (-2.14 to 3.71)	0.5969	0.64 (-2.67 to 3.94)	0.7056	0.90 (-2.89 to 4.68)	0.6417
**Outcome**	**Unadjusted mean difference in change from baseline (95% CI)**	**p-value**	**Adjusted mean difference in change from baseline (95% CI)**	**p-value**	**Adjusted mean difference in change from baseline (95% CI)**	**p-value**
SSQ-14 score						
Month 3	-1.65 (-3.07 to -0.24)	0.0219	-1.65 (-3.13 to -0.16)	0.0295	-1.60 (-3.08 to -0.11)	0.0347
Month 6	-1.57 (-2.98 to -0.15)	0.0302	-1.56 (-3.05 to -0.07)	0.0396	-1.51 (-3.00 to -0.02)	0.0466
Month 9	-1.63 (-3.05 to -0.22)	0.0233	-1.63 (-3.11 to -0.15)	0.0313	-1.58 (-3.06 to -0.09)	0.0374
Month 12	-0.78 (-2.19 to 0.63)	0.2799	-0.77 (-2.25 to 0.71)	0.3068	-0.72 (-2.20 to 0.76)	0.3399
PHQ-9 score						
Month 3	-0.35 (-1.68 to 0.99)	0.6130	-0.49 (-1.82 to 0.85)	0.4734	-0.47 (-1.82 to 0.88)	0.4963
Month 6	0.01 (-1.33 to 1.34)	0.9926	-0.14 (-1.47 to 1.20)	0.8412	-0.12 (-1.47 to 1.23)	0.8633
Month 9	-0.04 (-1.39 to 1.30)	0.9484	-0.19 (-1.53 to 1.15)	0.7847	-0.17 (-1.52 to 1.18)	0.8062
Month 12	0.74 (-0.60 to 2.08)	0.2778	0.60 (-0.74 to 1.93)	0.3792	0.62 (-0.73 to 1.96)	0.3690
**Outcome**	**Unadjusted odds ratio (95% CI)**	**p-value**	**Adjusted odds ratio (95% CI)**	**p-value**	**Adjusted odds ratio (95% CI)**	**p-value**
SSQ-14 ≥9						
Month 3	0.38 (0.09 to 1.59)	0.1857	0.28 (0.07 to 1.12)	0.0720	0.28 (0.07 to 1.09)	0.0668
Month 6	0.41 (0.09 to 1.83)	0.2453	0.30 (0.07 to 1.30)	0.1085	0.30 (0.07 to 1.27)	0.1026
Month 9	0.34 (0.07 to 1.59)	0.1701	0.25 (0.05 to 1.13)	0.0716	0.25 (0.06 to 1.10)	0.0668
Month 12	1.54 (0.33 to 7.15)	0.5778	1.12 (0.25 to 5.01)	0.8797	1.14 (0.26 to 4.88)	0.8647
PHQ-9 ≥11						
Month 3	3.24 (0.60 to 17.46)	0.1709	2.16 (0.47 to 9.98)	0.3260	1.86 (0.49 to 7.05)	0.3620
Month 6	3.17 (0.47 to 21.37)	0.2369	2.10 (0.35 to 12.47)	0.4137	1.84 (0.37 to 9.03)	0.4524
Month 9	3.84 (0.49 to 30.30)	0.2014	2.55 (0.37 to 17.74)	0.3445	2.24 (0.39 to 13.04)	0.3673
Month 12	3.11 (0.47 to 20.49)	0.2383	2.06 (0.36 to 11.85)	0.4164	1.81 (0.38 to 8.57)	0.4568
Viral load <1000 copies/mL						
Month 6	2.20 (0.79 to 6.14)	0.1309	2.26 (0.79 to 6.45)	0.1274	2.93 (0.73 to 11.79)	0.1297
Month 12	1.60 (0.42 to 6.05)	0.4850	1.75 (0.47 to 6.49)	0.3989	3.41 (0.40 to 29.30)	0.2603

MEMS=Medication Event Monitoring System, SSQ= Shona Symptoms Questionnaire, PHQ=Patient Health Questionnaire, CI=confidence interval

© 2023 Haas AD et al. *JAMA Network Open.*

eTable 2. Effect of the Friendship Bench Intervention on Adherence, Viral Load, and Mental Health: Results From Prespecified and Post Hoc Sensitivity Per-Protocol Analyses

	Prespecified analyses				Sensitivity analyses (post hoc)	
**Outcome**	**Unadjusted mean difference (95% CI)**	**p-value**	**Adjusted mean difference (95% CI)**	**p-value**	**Adjusted mean difference (95% CI)**	**p-value**
MEMS adherence						
Months 2-6	2.34 (-0.81 to 5.50)	0.1458	1.97 (-1.53 to 5.47)	0.2701	2.23 (-1.80 to 6.27)	0.2780
Months 1-12	1.35 (-1.60 to 4.30)	0.3699	0.95 (-2.37 to 4.27)	0.5739	1.22 (-2.63 to 5.07)	0.5358
**Outcome**	**Unadjusted mean difference in change from baseline (95% CI)**	**p-value**	**Adjusted mean difference in change from baseline (95% CI)**	**p-value**	**Adjusted mean difference in change from baseline (95% CI)**	**p-value**
SSQ-14 score						
Month 3	-1.72 (-3.13 to -0.31)	0.0166	-1.70 (-3.18 to -0.23)	0.0237	-1.64 (-3.11 to -0.16)	0.0300
Month 6	-1.60 (-3.01 to -0.19)	0.0259	-1.58 (-3.06 to -0.11)	0.0356	-1.52 (-2.99 to -0.04)	0.0444
Month 9	-1.72 (-3.12 to -0.31)	0.0166	-1.70 (-3.17 to -0.23)	0.0238	-1.63 (-3.11 to -0.15)	0.0303
Month 12	-0.85 (-2.25 to 0.56)	0.2374	-0.83 (-2.30 to 0.64)	0.2698	-0.76 (-2.23 to 0.71)	0.3118
PHQ-9 score						
Month 3	-0.36 (-1.71 to 0.98)	0.5977	-0.48 (-1.83 to 0.86)	0.4793	-0.47 (-1.82 to 0.89)	0.5015
Month 6	0.02 (-1.34 to 1.37)	0.9787	-0.10 (-1.45 to 1.25)	0.8806	-0.08 (-1.45 to 1.28)	0.9027
Month 9	-0.09 (-1.45 to 1.26)	0.8927	-0.22 (-1.57 to 1.14)	0.7549	-0.20 (-1.56 to 1.16)	0.7770
Month 12	0.66 (-0.69 to 2.01)	0.3361	0.54 (-0.80 to 1.88)	0.4312	0.56 (-0.79 to 1.91)	0.4179
**Outcome**	**Unadjusted odds ratio (95% CI)**	**p-value**	**Adjusted odds ratio (95% CI)**	**p-value**	**Adjusted odds ratio (95% CI)**	**p-value**
SSQ-14 ≥9						
Month 3	0.34 (0.08 to 1.43)	0.1412	0.25 (0.06 to 1.05)	0.0576	0.25 (0.06 to 1.01)	0.0522
Month 6	0.42 (0.10 to 1.84)	0.2500	0.31 (0.07 to 1.35)	0.1187	0.31 (0.07 to 1.32)	0.1128
Month 9	0.33 (0.07 to 1.55)	0.1592	0.24 (0.05 to 1.13)	0.0715	0.25 (0.05 to 1.10)	0.0666
Month 12	1.50 (0.32 to 6.93)	0.6039	1.11 (0.24 to 5.00)	0.8960	1.13 (0.26 to 4.88)	0.8749
PHQ-9 ≥11						
Month 3	3.21 (0.57 to 18.06)	0.1864	2.21 (0.46 to 10.70)	0.3235	1.98 (0.51 to 7.75)	0.3249
Month 6	3.24 (0.47 to 22.40)	0.2330	2.23 (0.37 to 13.54)	0.3823	2.04 (0.41 to 10.19)	0.3860
Month 9	3.75 (0.46 to 30.90)	0.2192	2.58 (0.35 to 18.82)	0.3495	2.37 (0.39 to 14.30)	0.3454
Month 12	2.68 (0.39 to 18.28)	0.3140	1.85 (0.31 to 10.95)	0.4997	1.69 (0.34 to 8.33)	0.5213
Viral load <1000 copies/mL						
Month 6	2.58 (0.89 to 7.53)	0.0817	2.52 (0.85 to 7.45)	0.0938	2.89 (0.72 to 11.65)	0.1356
Month 12	1.99 (0.46 to 8.53)	0.3529	2.05 (0.49 to 8.65)	0.3268	3.34 (0.36 to 30.70)	0.2850

MEMS=Medication Event Monitoring System, SSQ= Shona Symptoms Questionnaire, PHQ=Patient Health Questionnaire, CI=confidence interval

© 2023 Haas AD et al. *JAMA Network Open.*

eTable 3. Number of Participants With Safety-Relevant Outcomes by Trial Group

	**Friendship Bench** N=244	**Standard of Care** N=272
Reported actual or attempted self-harm	11 (4.5%)	5 (1.8%)
Before or on baseline	9 (3.7%)	5 (1.8%)
Incident event after baseline	2 (0.8%)	0 (0.0%)
Psychiatric hospitalizations	0 (0.0%)	0 (0.0%)
Deaths		
Unrelated to study procedures and intervention	5 (2.0%)	1 (0.4%)
Related to study procedures or intervention	0 (0.0%)	0 (0.0%)
Suspected or reported suicide	0 (0.0%)	0 (0.0%)

© 2023 Haas AD et al. *JAMA Network Open.*

eFigure. Forest Plot of Available Evidence of the Effect of the Friendship Bench Intervention on SSQ-14 Scores, PHQ-9 Scores, and Viral Suppression From 3 Trials

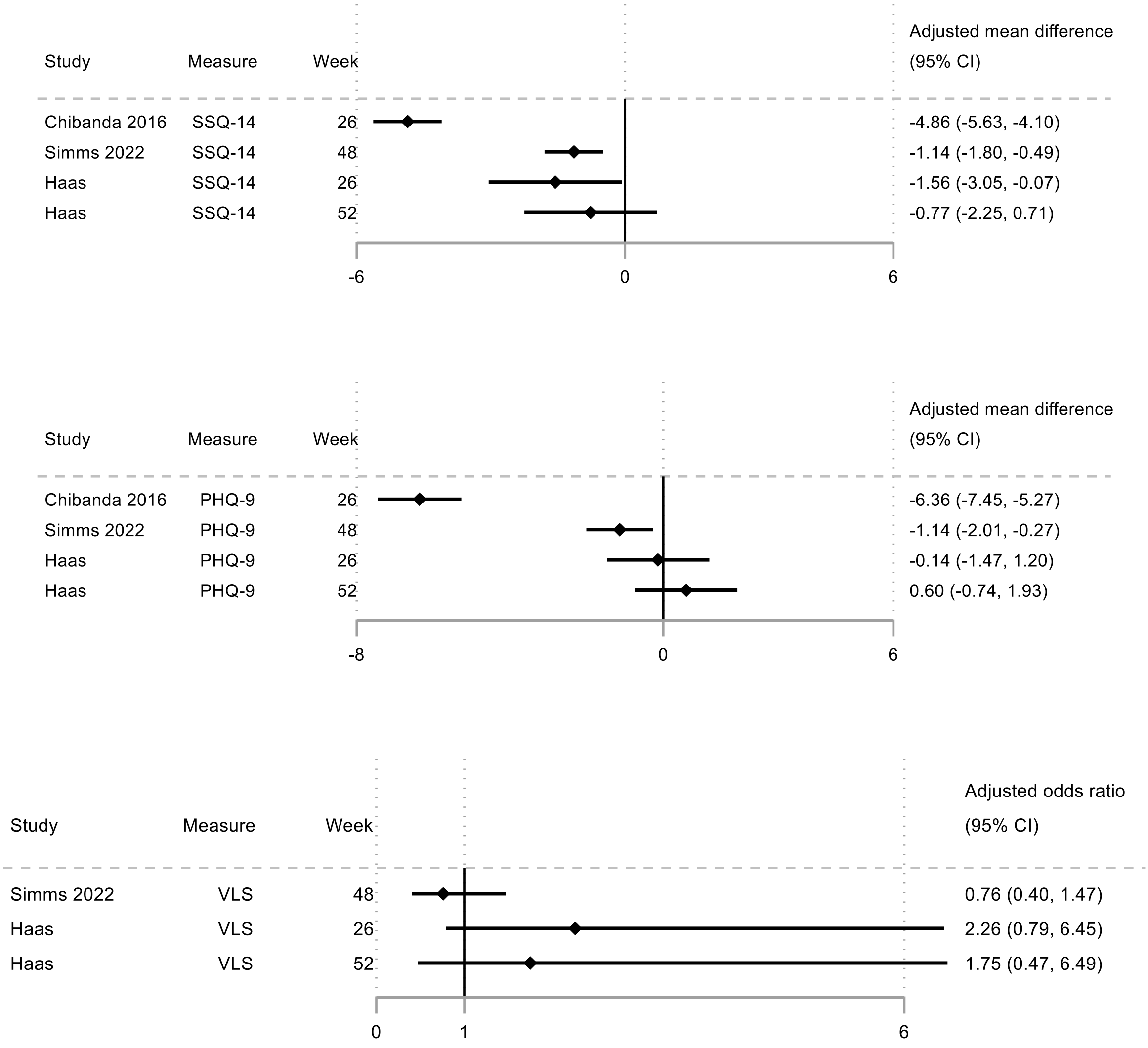

SSQ=Shona Symptoms Questionnaire, PHQ= Patient Health Questionnaire, VLS=viral suppression

© 2023 Haas AD et al. *JAMA Network Open.*
