# Supplementary material for: Effect of the Friendship Bench Intervention on Antiretroviral Therapy Outcomes and Mental Health Symptoms in Rural Zimbabwe: A Cluster Randomized Trial
Source: JAMA Netw Open. 2023 Jul 13;6(7):e2323205. doi: 10.1001/jamanetworkopen.2023.23205 (PMC10346120; doi:10.1001/jamanetworkopen.2023.23205)
Supplement: Supplement 3. — Nonauthor Collaborators [file jamanetwopen-e2323205-s003.pdf]

Supplemental Online Content: Nonauthor Collaborators
*First name, last name, and suffix (if applicable) are required and will appear in PubMed.

*Group Name(s): Friendship Bench -ART trial group							
***First Name and Middle Initial(s)**	***Last Name**	***Suffix (eg, Jr, III)**	**Academic Degrees**	**Institution**	**Location (city, state/province, country)**	**Role or Contribution, eg, chair, principal investigator**	**Group (if more than 1 Group listed in the byline) and/or Subgroup (eg, Steering Committee)**
Amos	Kateta		BSc, MSc	SolidarMed Zimbabwe	Masvingo	Research Assistant	
Cuthbert	Mutonhodza		MSc	SolidarMed Zimbabwe	Masvingo	Research Assistant	
Favourite	Machiha		BA	SolidarMed Zimbabwe	Masvingo	Research Assistant	
Ntandoyenkosi	Mhlanga		BSc	SolidarMed Zimbabwe	Masvingo	Research Assistant	
Shingai	Matutururu		MSc	SolidarMed Zimbabwe	Masvingo	Research Assistant	
Tatenda	Gombwiro		BSc	SolidarMed Zimbabwe	Masvingo	Research Assistant	
Millicent	Gweredza		BA	SolidarMed Zimbabwe	Masvingo	Research Assistant	
Josephine Yasmin	Saide		MSc	SolidarMed Zimbabwe	Masvingo	Research Assistant	
Beauty	Muchakubvura		MSc	SolidarMed Zimbabwe	Masvingo	Research Assistant	
Kudzai	Mhlanga		MPA	SolidarMed Zimbabwe	Masvingo	Research Assistant	
Dennis	Mwakasa		BA	SolidarMed Zimbabwe	Masvingo	Research Assistant	
Fungai Nyikadzino	Zvekare		MSc	SolidarMed Zimbabwe	Masvingo	Research Assistant	
Yvonne Chiedza	Maumbe		BSc, MPH	SolidarMed Zimbabwe	Masvingo	Research Assistant	
Boldwin	Maposa		BSc	SolidarMed Zimbabwe	Masvingo	Research Assistant	
Sibongile	Gumbo		MSc	SolidarMed Zimbabwe	Masvingo	Research Assistant	
Japhet	Kamusha		MSc	SolidarMed Zimbabwe	Masvingo	Research Assistant	
Morris	Tshuma		BSc, MSc	SolidarMed Zimbabwe	Masvingo	Research Assistant	
Rejoyce	Runyowa		MSc, BSc	SolidarMed Zimbabwe	Masvingo	Research Assistant	
Abigail	Pikayi		MSc, BSc, BA	SolidarMed Zimbabwe	Masvingo	Research Assistant	
Fredrick	Mbiba		MSc	SolidarMed Zimbabwe	Masvingo	Research Assistant	
Chiedza Elizabeth	Mutungama		MSc, BSc	SolidarMed Zimbabwe	Masvingo	Research Assistant	
Waraidzo AM	Mukuwapasi		MSc	SolidarMed Zimbabwe	Masvingo	Research Assistant	
Rudo	Mandabva		BSc	SolidarMed Zimbabwe	Masvingo	Research Assistant	
Sven	Trelle		MD	CTU Bern, University of Bern	Bern, Switzerland	Study design	
Ethel	Manda		BSc	Friendship Bench	Harare	Friendship Bench trainer	
Duncan Tatenda	Majichi		MSc	Friendship Bench	Harare	Friendship Bench trainer	
