# Supplementary material for: Effect of the Friendship Bench Intervention on Antiretroviral Therapy Outcomes and Mental Health Symptoms in Rural Zimbabwe: A Cluster Randomized Trial
Source: JAMA Netw Open. 2023 Jul 13;6(7):e2323205. doi: 10.1001/jamanetworkopen.2023.23205 (PMC10346120; doi:10.1001/jamanetworkopen.2023.23205)
Supplement: Supplement 4. — Data Sharing Statement [file jamanetwopen-e2323205-s004.pdf]

Data Sharing Statement

Haas. Effect of the Friendship Bench Intervention on Antiretroviral Therapy Outcomes and Mental Health Symptoms in Rural Zimbabwe. *JAMA Netw Open*. Published July 13, 2023. doi:10.1001/jamanetworkopen.2023.23205

Data
Data available: No

Additional Information
Explanation for why data not available: Data cannot be made available online because of legal and ethical restrictions. To request data, readers may contact IeDEA-SA for consideration.
